# Cannabidiol (CBD) as a Promising Anti-Cancer Drug

**DOI:** 10.3390/cancers12113203

**Published:** 2020-10-30

**Authors:** Emily S. Seltzer, Andrea K. Watters, Danny MacKenzie, Lauren M. Granat, Dong Zhang

**Affiliations:** 1 Department of Biomedical Sciences, College of Osteopathic Medicine, New York Institute of Technology, Old Westbury, NY 11568, USA; eseltz01@nyit.edu (E.S.S.); awatters@nyit.edu (A.K.W.); dmackenz@nyit.edu (D.M.J.); 2 Department of Internal Medicine, Cleveland Clinic, Cleveland, OH 44195, USA; GranatL2@ccf.org

**Keywords:** cannabinoids, cannabidiol, CBD, anti-cancer drug

## Abstract

**Simple Summary:**

The use of cannabinoids containing plant extracts as herbal medicine can be traced back to as early as 500 BC. In recent years, the medical and health-related applications of one of the non-psychotic cannabinoids, cannabidiol or CBD, has garnered tremendous attention. In this review, we will discuss the most recent findings that strongly support the further development of CBD as a promising anti-cancer drug.

**Abstract:**

Recently, cannabinoids, such as cannabidiol (CBD) and Δ^9^-tetrahydrocannabinol (THC), have been the subject of intensive research and heavy scrutiny. Cannabinoids encompass a wide array of organic molecules, including those that are physiologically produced in humans, synthesized in laboratories, and extracted primarily from the *Cannabis sativa* plant. These organic molecules share similarities in their chemical structures as well as in their protein binding profiles. However, pronounced differences do exist in their mechanisms of action and clinical applications, which will be briefly compared and contrasted in this review. The mechanism of action of CBD and its potential applications in cancer therapy will be the major focus of this review article.

## 1. Introduction

**Figure 1:**
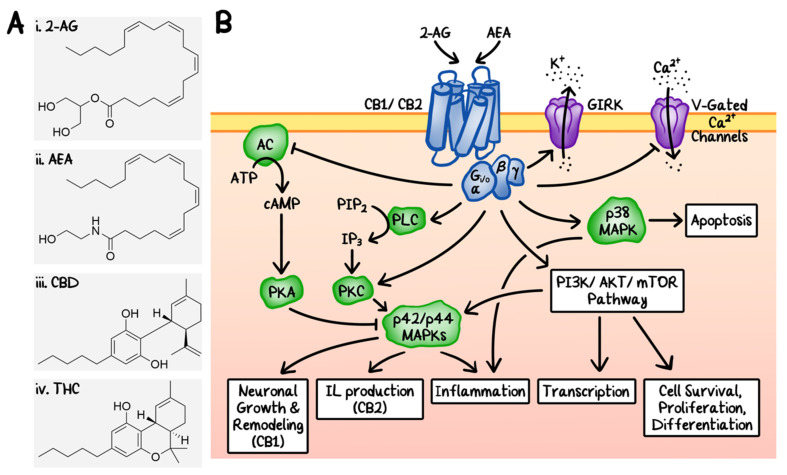
Endocannabinoid system. (**A**) Chemical structures of two endogenous cannabinoids, 2-arachidonylglycerol (**i**, 2-AG) and *N*-arachidonylethanolamine (**ii**, AEA), and two representative exogenous cannabinoids from *Cannabis sativa*, cannabidiol (**iii**, CBD) and Δ^9^-tetrahydrocannabinol (**iv**, Δ^9^-THC). (**B**) Schematic diagrams of the signaling transduction pathways of the endocannabinoid system. 2-AG and AEA are agonists of CB_1_ and CB_2_. Some of the downstream effects include: (1) upregulation of p42/p44 mitogen-activated protein kinases (MAPKs) by direct inhibition of adenylyl cyclase (AC) and direct activation of phospholipase C (PLC), leading to the induction of neuronal growth, interleukin production, and inflammation. PKA: protein kinase A. PKC: protein kinase C. (2) Activation of p38 MAPK, which induces inflammation and apoptosis. (3) Activation of the phosphatidylinositol-3-kinase (PI3K)/AKT and the mammalian target of rapamycin (mTOR) signaling pathways. Under certain conditions, these endocannabinoids can also induce transcription, cell survival, proliferation, and differentiation through similar pathways. Additionally, the cannabinoid receptors can also modulate ion channels including G protein-coupled inwardly-rectifying potassium channels (GIRKs) and voltage (V)-gated calcium channels.

The use of *Cannabis sativa* plant extract as herbal medicine can be dated back as early as 500 BC in Asia. The human endocannabinoid system was uncovered after the discovery of the cannabinoid receptors [1]. It was initially thought that cannabinoids produce their physiological effects via non-specific interactions with the cellular membrane; however, research involving rat models in the late-1980s led to the discovery and characterization of specific cannabinoid receptors, CB_1_ and CB_2_ [2,3]. The CB_1_ receptor is expressed throughout the central nervous system (CNS), whereas the CB_2_ receptor is found primarily in the immune system and hematopoietic cells [4]. Soon after the discovery of CB_1_ and CB_2_, their endogenous ligands, or endocannabinoids, were also identified, including 2-arachidonolyglycerol (2-AG) and *N*-arachidonoylethanolamine (AEA, also called anandamide) (Figure 1A, i and ii) [5,6,7,8]. CB_1_ and CB_2_ belong to a large family of transmembrane proteins, called G protein-coupled receptors (GPCRs), and are now believed to be responsible for the majority of the physiological effects of the endocannabinoids (Figure 1B). Both receptors are coupled with Gα_i/o_, which can inhibit the adenylyl cyclase (AC) [4,9]. CB_1_ can also be coupled to Gα_q/11_ [10] and Gα_12/13_ [11]. CB_2_ has also been shown to act through Gα_s_ [12]. For a more in-depth understanding of the downstream effects of the endocannabinoids and their receptors under physiological conditions, we refer you to other excellent reviews on the topic [13,14].

The two primary endocannabinoids, 2-AG and AEA, can activate either CB_1_ or CB_2_ and are synthesized on-demand from phospholipid precursors in response to an elevation of intracellular calcium [15,16]. In addition to CB_1_ and CB_2_, 2-AG and AEA can also bind other transmembrane proteins, including orphan G protein-coupled receptor 55 (GPR55), peroxisome proliferator-activated receptors (PPARs), and transient receptor potential vanilloid (TRPV) channel type 1 (TRPV1) [17,18].

The TRPV channels are of particular interest concerning the anti-tumor functions of cannabidiol (CBD) (Figure 1A, iii), which will be discussed in more detail later. Six different TRPV channels have been identified in humans and can be subdivided into two groups: TRPV1, TRPV2, TRPV3, and TRPV4 belong to group I, while TRPV5 and TRPV6 fall into group II [19]. Though the exact functions of the TRPV channels are still under intense investigation, they are likely involved in regulating cellular calcium homeostasis. For example, TRPV1 and TRPV2 can be found in the cytoplasmic membrane as well as the endoplasmic reticulum (ER) membrane. They both play an important role in regulating the cytoplasmic calcium concentration from the extracellular sources as well as the calcium stored within the ER. Disruption of cellular calcium homeostasis can lead to increased production of reactive oxygen species (ROS), ER stress, and cell death.

A variety of cannabinoids exist in the *Cannabis sativa* plant (also known as the hemp or marijuana plant). There are more than 100 different cannabinoids and Δ^9^–tetrahydrocannabinol (Δ^9^-THC) (Figure 1A, iv) and CBD are the most well-known ones. The so called drug-type *Cannabis sativa* contains higher level of Δ^9^-THC and is used more widely for medical and recreational purposes, whereas the fiber-type cannabis contains less than 0.2% of Δ^9^-THC and is more often used in textiles and food [20,21]. Δ^9^-THC is thought to be the psychotic cannabinoid and many of its psychoactive effects are due to its interaction with the CB_1_ receptor, whereas its immune-modulatory properties are likely due to its interaction with the CB_2_ receptor. In contrast, CBD is non-psychoactive and has a relatively low affinity to both CB_1_ and CB_2_ [22].

The utility of cannabinoids in the treatment of cancer has long been of great interest. Recently, both CB_1_ and CB_2_ were found to be expressed in many cancer types. Intriguingly, both receptors were often undetectable at the site of the cancers’ origin before neoplastic transformation [23]. Additional evidence for the role of endocannabinoid system in neoplasia came when Wang and colleagues showed that CB_1_ has a tumor-suppressive function in a genetically modified mouse model of colon cancer [24]. On the other hand, CB_1_ is upregulated in hepatocellular carcinoma and Hodgkin lymphoma, and the extent to which CB_1_ was overexpressed correlated with disease severity in epithelial ovarian carcinoma [25,26,27]. Similarly, CB_2_ has also been found to be overexpressed in HER2+ breast cancers and gliomas [28,29]. Finally, it was shown that overexpression of both CB_1_ and CB_2_ was correlated with poor prognosis in stage IV colorectal carcinoma [30,31]. In 1976, Carchman and colleagues found that the administration of cannabinoids, such as Δ^8^-THC, Δ^9^-THC, and CBD, inhibited the DNA synthesis and growth of lung adenocarcinoma in cultured cells as well as mouse tumor models [32,33]. Similar effects were seen in both in vitro and in vivo models of various other cancers, including glioma, breast, pancreas, prostate, colorectal carcinoma, and lymphoma [34,35]. There are various proposed mechanisms of action behind these findings, including, but not limited to: cell cycle arrest, induction of apoptosis, as well as inhibition of neovascularization, migration, adhesion, invasion, and metastasis [36]. Despite the multitude of positive results with Δ^9^-THC-related cannabinoids in cancer research, the clinical use of these compounds is limited due to their psychoactive side effects.

In contrast to the Δ^9^-THC-related cannabinoids, CBD has no known psychoactive effects, and therefore, has recently been the focus of intense research in many therapeutic areas, including cancer. At present, the Food and Drug Administration (FDA) has only approved Epidiolex, purified CBD, for use in patients with seizures associated with the Lennox-Gastaut syndrome or Dravet syndrome [37]. Globally, more than 40 countries have approved medical marijuana/cannabis programs, whereas this is true of 34 states in the USA, plus the District of Columbia, Guam, Puerto Rico, and US Virgin Islands. While marijuana is considered a Schedule I controlled substance in the US, the Drug Enforcement Administration ruled that CBD is a Schedule V controlled substance [38]. When approved by the FDA, CBD must contain less than 0.1% of Δ^9^-THC.

It has been noted that CBD has a relatively low affinity to both CB_1_ and CB_2_ [22]. However, it was found that CBD can act as an antagonist to CB_1_ in the mouse vas deferens and brain tissues in vitro [39]. There is also evidence suggesting that CBD may act as an inverse agonist of human CB_2_ [22]. Other cellular receptors that CBD may interact with include TRPVs, 5-HT1A, GPR55, and PPARγ [40]. It has been hypothesized that CBD has robust anti-proliferative and pro-apoptotic effects. In addition, it may also inhibit cancer cell migration, invasion, and metastasis [1]. The utility of CBD in anti-tumor therapy and the potential mechanisms behind it will be discussed in more detail below. Since much of the anti-tumor activity of CBD seems to hinge on its regulation of ROS, ER stress, and immune modulation, we will first summarize the interplays between ROS, ER stress, and inflammation and their known effects on various aspects of tumorigenesis. Thereafter, we will further discuss the anti-tumor effects of CBD on a variety of cancers and the molecular mechanisms behind them.

## 2. The Interplays between Reactive Oxygen Species (ROS), ER Stress, Inflammation, and Cancers

### 2.1. ROS and Cancers

**Figure 2:**
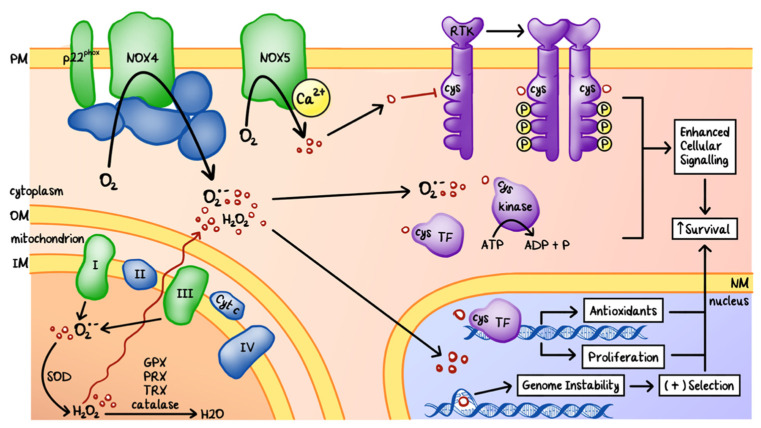
Origins and effects of cellular reactive oxygen species (ROS). ROS are generated by complex I and III of the electron transport chain in the mitochondria and by NADPH oxidase (NOX) enzymes located at the cytoplasmic membrane (PM). ROS disrupt cellular processes by oxidizing the cysteine (Cys) residues of various proteins and damaging nucleic acids. Oxidation by ROS could cause the inactivation of phosphatases, activation of kinases and transcription factors (TF), and genomic alterations, leading to enhanced cellular proliferation and survival. ROS production is counteracted by the generation of antioxidants, such as superoxide dismutase (SOD), glutathione peroxidase (GPX), peroxiredoxin (PRX), thioredoxin (TRX), and catalase. In cancers, redox homeostasis is modified to favor ROS tolerance. OM: outer mitochondrial membrane. IM: inner mitochondrial membrane. NM: nuclear membrane.

ROS refer to various oxygen-containing species that are energetically unstable and highly reactive with a variety of biomolecules, including amino acids, lipids and nucleic acids. Commonly seen ROS include superoxide (O_2_^−^), peroxide (O_2_^−2^), hydrogen peroxide (H_2_O_2_), and hydroxyl free radical (OH^−^) [41,42,43,44]. The most common sources of ROS are the electron transport chain in the mitochondria and the NADPH oxidase (NOX) family of transmembrane enzymes (Figure 2). Certain enzymes and organelles, such as peroxisomes and ER, can also produce ROS. ROS can directly oxidize nucleic acids, proteins, and lipids thus altering or disrupting their functions [45].

To prevent constant damage to biomolecules, ROS are counter-balanced by various antioxidants inside the cells. Major anti-oxidant enzymes include superoxide dismutase (SOD), catalase, peroxiredoxin (PRX), thioredoxin (TRX), and glutathione peroxidase (GPX) [42].

In cancers, the redox balance is altered so that increased ROS production favors tumor progression and expansion while evading cell death. The pro-tumor effects of increased ROS generation include, but are not limited to, genomic instability and enhanced proliferation [42,43,44] (Figure 2). ROS damage DNA by oxidizing guanine and forming 8-hydroxyguanine and 8-nitroguanine. This could lead to deletions/insertions, mutations in base pairing, and strand breaks followed by mutagenic repair [44,45]. Genome instability plays a key role in tumor progression through the accumulation of mutations that promote uncontrolled growth and evade cell death [43]. Proliferation is further enhanced through the oxidation and activation of the pro-growth intracellular signaling pathways, including mitogen-activated protein kinase (MAPK) pathways and the phosphatidylinositol-3-kinase (PI3K)/protein kinase B (AKT) pathway. Nuclear factor kappa-light-chain-enhancer of activated B cells (NF-κB), a transcription factor vital for growth and migration, also becomes activated by ROS through inhibiting the phosphorylation of the inhibitor of NF-κB α (IκBα), or through promoting the S-glutathionylation of the inhibitor of NF-κB kinase subunit β (IKKβ). Finally, cancer cells can rewire their signaling transduction pathways to cope with elevated intracellular ROS. Most notably, this can be achieved through increased mitochondrial SOD activity or inactivation of the scavenging enzymes [42,46].

Nonetheless, toxic levels of ROS can induce cell death or autophagy in cancer cells. ROS modulate calcium channels, pumps, and exchangers activity by oxidizing their Cys residues [43]. The increase of intracellular mitochondrial calcium or the oxidation of lipids damages the mitochondrial membrane resulting in the release of cytochrome c, a potent activator of apoptosomes [42,45]. ROS can also directly affect caspase activity and cleavage of Bcl-2, and/or increase the expression of cell death receptors such as TRAIL and Fas [47]. Autophagy can be induced by the activation of the mTOR pathway.

### 2.2. Endoplasmic Reticulum (ER) Stress and Cancers

ER is an important organelle that plays a critical role in post-translational modification and folding of proteins, calcium homeostasis, and other biological processes [48,49]. Accumulation of unfolded and/or misfolded proteins triggers the unfolded protein response (UPR), which helps to re-balance the ER homeostasis. UPR temporarily halts protein synthesis and attempts to correct and re-fold proteins. In the case that the unfolded and/or misfolded proteins cannot be corrected in time, they will then be targeted for protein degradation.

**Figure 3:**
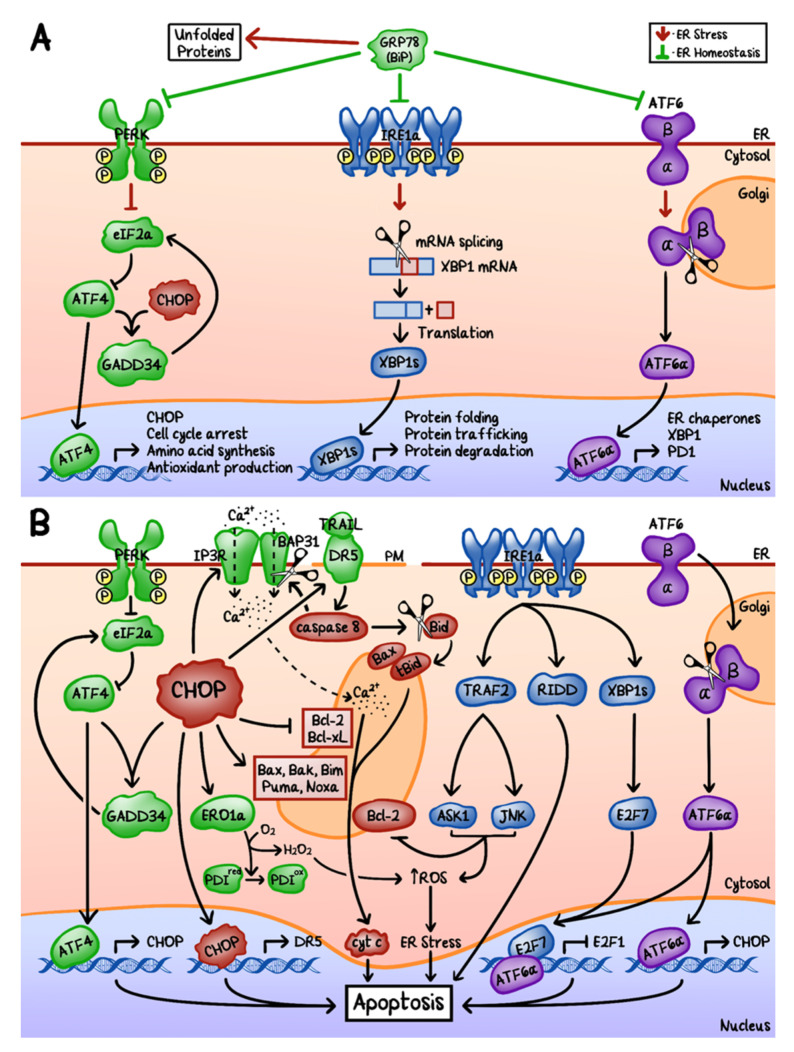
Endoplasmic reticulum (ER) homeostasis, stress, and the unfolded protein response (UPR). (**A**) ER homeostasis is mediated by 78-kDa glucose-regulated protein (GRP78). Under stress conditions, GRP78 dissociates from pancreatic endoplasmic reticulum kinase (PERK), inositol-requiring enzymes 1α (IRE1α), as well as the activating transcription factor 6 (ATF6), leading to activation of their downstream signaling cascades in order to restore ER homeostasis. (**B**) When ER homeostasis fails to be restored, excessive UPR could lead to apoptosis, primarily via upregulation of C/EBP homologous protein (CHOP). PM: cytoplasmic membrane; eIF2α: eukaryotic initiation factor 2α; ATF4: activating transcription factor 4; GADD34: DNA damage inducible protein 34; XPB1: X-box-binding protein (XBP1s: spliced form); ERO1α: endoplasmic reticulum oxidoreductase 1α; PDI: protein disulfide isomerase; DR5: death receptor 5; TRAIL: TNF related apoptosis-inducing ligand; IP3R: inositol 1,4,5-triphosphate receptor; BAP31: B cell receptor-associated protein 31; Bid: BH3 Interacting Domain Death Agonist; TRAF2: tumor necrosis factor receptor-associated factor 2; RIDD: regulated IRE1-dependent decay; ASK1: apoptosis signal-regulating kinase 1; JNK: JUN N-terminal kinase; E2F7: E2F transcription factor 7; E2F1: E2F transcription factor 1.

UPR is a well-studied cellular process (Figure 3A). It is primarily regulated by the 78-kDa glucose-regulated protein (GRP78), which is also known as the binding immunoglobulin protein (BiP) [49]. Under non-stress conditions, GRP78 binds and inhibits three transmembrane proteins: inositol-requiring enzymes 1α (IRE1α), pancreatic endoplasmic reticulum kinase (PERK), as well as the activating transcription factor 6 (ATF6) [48,49]. Whereas under ER stress conditions, GRP78 binds the unfolded proteins, dissociates from PERK, IRE1α, and ATF6, and results in the activation of three distinct, but interconnecting, pathways. Downstream of the PERK and ATF6 cascades, CHOP activity is increased.

CHOP induces apoptosis via multiple pathways (Figure 3B): (i) It increases the transcription of GADD34 [49]; (ii) It increases the transcription of ER oxidoreductase 1 alpha (ERO1α), which then re-oxidizes PDI and generates ROS; (iii) It increases the transcription of the inositol 1,4,5-triphosphate receptor (IP3R), which then increases the calcium level in the cytoplasm; (iv) It activates the extrinsic cell death pathway via death receptor 5 (DR5) and caspase-8 mediated activation of truncated Bid (tBid), which then translocates to the mitochondria and promotes the release of cytochrome c; (v) It activates the intrinsic cell death pathway by directly decreasing the expression of pro-survival factors, Bcl-2 and Bcl-xL, and increasing the expression of pro-apoptotic factors, such as Bax, Bak, Bim, Puma, and Noxa; (vi) It activates caspase-8 via TRAIL-DR5 on the cytoplasmic membrane, which cleaves B cell receptor-associated protein 31 (BAP31) and forms p20. p20 then releases calcium from the ER into the cytoplasm, which is taken up by mitochondria and results in the further release of cytochrome c.

During their development, tumors rely heavily on the UPR pathway for cell survival, possibly due to the hypoxic environment and metabolic stress accompanying the rapidly increasing tumor mass. For example, PERK and ATF4 activate vascular endothelial growth factor (VEGF) and hypoxia-inducible factor 1/2 (HIF1/2) for angiogenesis [48]. The silencing of the XBP1 gene prevented tumor growth and metastasis of triple-negative breast cancer (TNBC) in vivo [50]. Analysis using TNBC cell lines demonstrated that the upregulation of XBP1 enhanced HIF1α expression. Nonetheless, when the URP system becomes overwhelmed, pro-apoptotic factors become dominant, leading to cell death.

### 2.3. The Effects of Inflammation and Microenvironment on Tumor Survival, Migration, and Immune Evasion

Tissue microenvironment often plays an important role in supporting tumor establishment, expansion, and metastasis. The tumor microenvironment is primarily comprised of infiltrated leukocytes, including tumor-associated macrophages (TAMs), dendritic cells, and myeloid-derived suppressor cells (MDSC) [51]. The crosstalk between the infiltrated cells and tumor cells could suppress the immune response and create a pro-survival environment for tumor cells.

**Figure 4:**
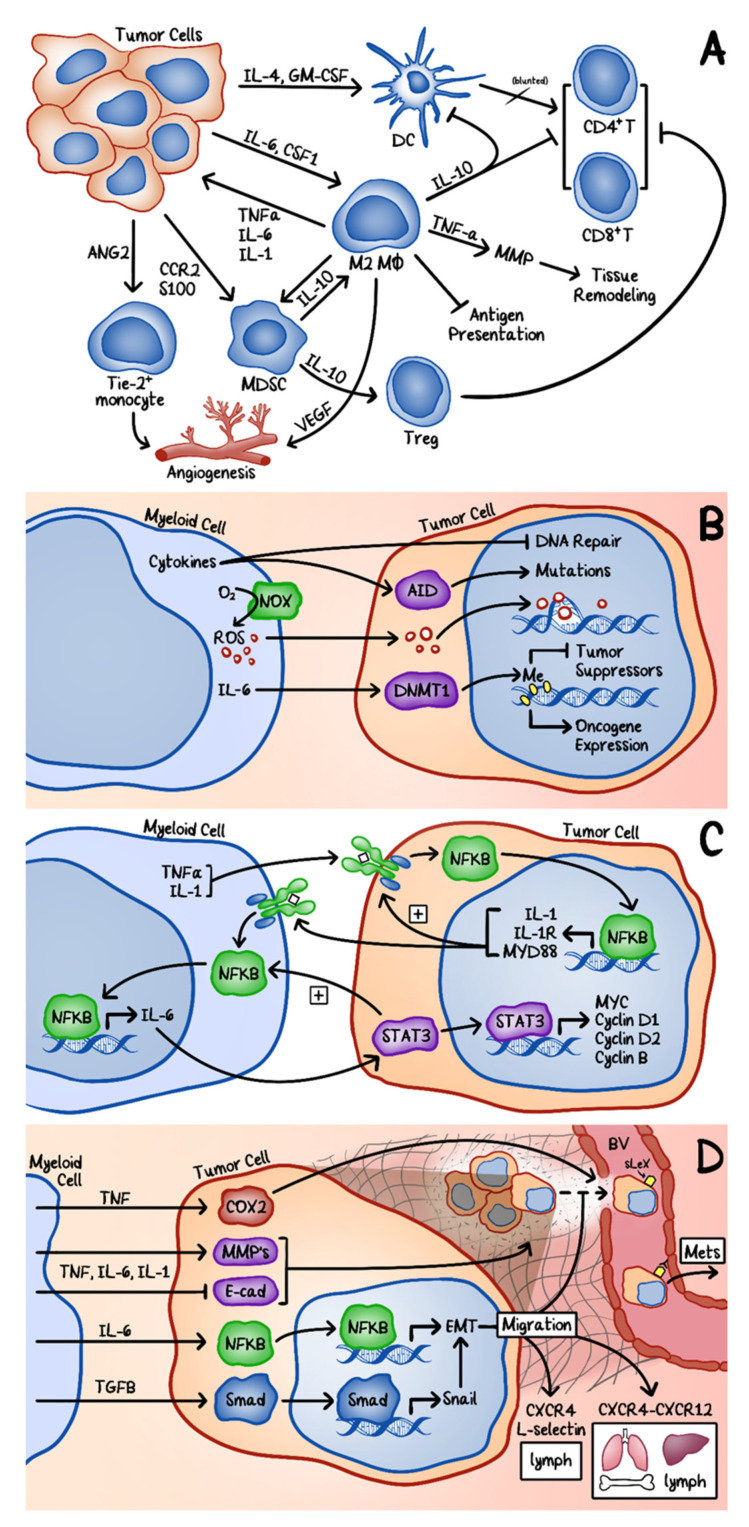
The interplays between tumor cells and inflammatory cells during tumorigenesis. (**A**) The effect of tumor cells on inflammatory cells. Tumor cells secrete many cytokines to alter the microenvironment to promote tumor growth and invasion and to blunt the anti-tumorigenic immune response. (**B**) Inflammatory cells affect the genomic stability of tumor cells. AID: activation-induced cytidine deaminase; DNMT1: DNA methyltransferase 1. (**C**) Inflammatory cells enhance tumor cell proliferation and survival through autocrine and paracrine signaling. (**D**) Inflammatory cells promote tumor cell migration, invasion, and metastasis through cytokine and chemokine production. COX-2: cyclooxygenase 2; MMP: matrix metalloproteinase; E-cad: E-cadherin; EMT: epithelial-mesenchymal transition; sLex: sialyl Lewis X; CXCR: CXC chemokine receptor; BV: blood vessel.

Evasion of the attack by the immune system is essential during the development of cancers. This is accomplished through dynamic interactions between different cytokines and their receptors in the tumor microenvironment. Tumors actively secrete different cytokines that attract a variety of infiltrating cells, such as TAMs, dendritic cells, MSDCs, and immunosuppressive regulatory T cells, which in turn help tumors to evade the attack by the immune system (Figure 4A). Cytokines released from myeloid cells can also induce genomic instability in tumor cells by directly damaging DNA or epigenetically altering the expression of genes (Figure 4B).

The key inflammatory mediators for tumor proliferation and survival include NF-κB and signal transducer and activator of transcription 3 (STAT3) (Figure 4C) [52]. IL-6, secreted by the myeloid cells, activates STAT3, which then upregulates cyclins D1, D2, and B as well as MYC to promote tumor cell proliferation. STAT3 expressed by the tumor cells further enhances IL-6 secretion by the myeloid cells via increased expression of NF-κB in these inflammatory cells, thus creating a positive feedback loop. IL-22, produced by the CD11c+ lymphoid cells, is also able to activate STAT3 in epithelial cells. In parallel, TNF-α and IL-1 secretion from leukocytes can upregulate the expression of NF-κB in tumor cells [52,53,54]. NF-κB, in turn, upregulates the expression of IL-1α, IL-1R, and MYD88, which can further enhance the activity of NF-κB, thus creating a positive autocrine loop [52]. The expression of NF-κB can be directly activated in immune cells by the inflammatory cytokines, TNF-α and IL-1, and by TLR-MYD88 from tissue damage [53,54]. Downstream of IL-6 signaling, NF-κB has also been shown to induce epithelial-mesenchymal transition (EMT), which then promotes tumor cell migration [54]. In a prostate cancer model, the interaction between receptor activator of NF-κB (RANK), on the surface of cancer cells, and RANK ligand, on the infiltrating leukocytes, promoted metastasis through the activation of NF-κB pathway. This NF-κB/IL-6/STAT3 positive feedback loop is present in all phases of tumorigenesis.

Furthermore, the expression of STAT3 in tumor-associated leukocytes also plays a key role in immune modulation. STAT3 expression in inflammatory cells allows for immune evasion of tumors, while STAT3 deletion in macrophages and neutrophils enhances Th1-mediated response with increased production of IFNγ, TNF-α, and IL-1 [55]. STAT3 expression in myeloid cells can inhibit the maturation of dendritic cells by downregulating their IL-12 expression and suppresses the immune response by upregulating the expression of IL-23 in TAMs [53].

Collectively, the activation of NF-κB and STAT3 signaling transduction pathways in cancer cells, as well as in the inflammatory cells in the tumor microenvironment, provide a great advantage for tumor cell proliferation, survival, migration, and immune evasion (Figure 4C,D).

## 3. The Anti-Cancer Effects of CBD

### 3.1. Glioma

Glioma is the most common primary brain malignancy. The grade IV glioma, also called glioblastoma multiforme (GBM) or glioblastoma, is one of the most aggressive types of cancer. The prognosis of GBM is very poor with only 4–5% survival within five years. Current treatment modalities include surgery, followed by radiotherapy and chemotherapy with Temozolomide (TMZ) or Carmustine (BCNU). Unfortunately, most GBM tumors are resistant to these treatments.

Cannabinoids have been studied to a great extent in gliomas due to the urgent unmet medical needs. The Table S1 summarizes the published studies focusing on CBD’s effects on gliomas either alone or together with BCNU, TMZ, tamoxifen, cisplatin, γ-irradiation, ATM inhibitors, and Δ^9^-THC [56,57,58,59,60,61,62,63,64]. In these studies, many GBM cell lines were used with a majority using U87MG [56,57,58,60,61,63,65,66,67,68,69,70,71,72,73,74]. The anti-proliferative effects of CBD on GBMs are quite clear, but the average IC_50_ values of CBD differed among different cell lines: C6 (8.5 µM) [67], U87MG (12.75 ± 9.7 µM), U373 (21.6 ± 3.5 µM) [65,75], U251 (4.91 ± 6.1 µM) [57,60], SF126 (1.2 µM) [57], T98 (8.03 ± 4.0 µM) [58,59,60,70,73], MZC (33.2 µM) [69], and GL261(10.67 ± 0.58 µM) [59]. Variation among different publications may be due to procedural differences, including assays used to measure the viability and/or time of CBD exposure.

CBD, alone or with other agents, has been shown to successfully induce cell death, inhibit cell migration and invasion in vitro, decrease tumor size, vascularization, growth, and weight, and increase survival and induce tumor regression in vivo [58,59,62,65,68,70,71,74]. Regarding CBD’s anti-proliferative action on GBM, data show that apoptosis occurs independent of CB_1_, CB_2_, and TRPV1, but is dependent on TRPV2 [58,65,66,67,69,72]. Specifically, Ivanov et al. found that CBD, γ-irradiation, and ATM inhibitor KU60019 upregulate TNF/ TNFR1 and TRAIL/ TRAIL-R2 signaling along with DR5 within the extrinsic apoptotic pathway [61,64]. CBD also activates the JNK-AP1 and NF-κB pathways to induce cell death. Less emphasis has been placed on the role of autophagy or cell cycle arrest in CBD-mediated effects on glial cells [57,58,64,72,74].

Many downstream effects of CBD have been investigated. Multiple papers reported an increased level of oxidative stress in CBD, but not Δ^9^-THC, treated GBM cell lines [58,65,73,76]. Massi et al. found that the level of ROS increases in a time-dependent manner, with significance after only five hours, when U87MG cells were treated with 25 µM CBD [76]. At the same time, glutathione, an antioxidant, was significantly decreased after six hours of CBD treatment. In contrast, there is no pronounced ROS increase in CBD treated normal glial cells. Co-treatment of CBD and antioxidants, including *N-*acetyl cysteine (NAC) and α-tocopherol (i.e., vitamin E), attenuated CBD’s killing effects [58]. Taken together, studies in GBM cell lines suggest that CBD induces cell death most likely by upregulating ROS. Scott et al. found that CBD also increased the expression of heat shock proteins (HSPs), which was found to be associated with the increased production of ROS because NAC hindered the role of HSPs [73]. Interestingly, the use of HSP inhibitors together with CBD were shown to increase the cytotoxic effect and reduce CBD’s IC_50_ value significantly, from 11 ± 2.7 µM to 4.8 ± 1.9 µM in T98G cells. This suggests that HSP inhibitors may be used as an adjunctive treatment with CBD. Recently, Aparicio-Blanco et al. administered CBD in lipid nanocapsules (LNCs) to GBM in vitro in an attempt to provide a prolonged-release formula of CBD [75]. LNCs loaded with CBD were more effective at decreasing the IC_50_ values when they were smaller in size and exposed for longer periods.

In GBMs, CBD inhibits the PI3K/AKT survival pathway by downregulating the phosphorylation of AKT1/2 (pAKT) and p42/44 MAPKs without effecting the total AKT and p42/44 MAPK protein levels [57,59,61,70,72,73]. This pathway may also be responsible for CBD-mediated autophagy in glioma stem-like cells, since in those cells, PTEN is upregulated while AKT is downregulated [72]. PI3K pathway plays an important role in the expression of TRPV2, which is a potential target of CBD. In U251, Δ^9^-THC and CBD together, but not separately, downregulated p42/44 MAPKs [57]. Whereas Scott et al. revealed that alone, CBD treated T98G and U87MG cells, albeit at a higher concentration (20 µM), decreased pAKT and p42/44 MAPKs levels, and more so when combined with γ-irradiation [59]. CBD can also activate the pro-apoptotic MAP kinase pathway. Ivanov et al. found that CBD treatment together with γ-irradiation led to the upregulation of active JNK1/2 and p38 MAPK, especially in U87MG cells [61]. However, using U251 cells, Marcu et al. showed that Δ^9^-THC and CBD did not increase the activity of JNK1/2 or p38 MAPK [57]. The discrepancy could be due to the genetic difference among different GBM cell lines.

Massi et al. explored how CBD modulates 5-lipoxygenase (5-LOX), COX-2, and the endocannabinoid system in GBMs [68,73,76]. They found that 5-LOX, but not COX-2, was decreased by CBD in vivo. CBD treatment also resulted in increased expression of fatty acid amide hydrolase (FAAH), which reduces the level of AEA, suggesting that CBD may inhibit the production of inflammatory mediators by indirectly attenuating the endocannabinoid system in GBMs.

In addition to γ-irradiation, CBD has also been tested with alkylating agents, especially TMZ, proving together to have synergistic anti-proliferative effects on glioma cells [60,62,63,74]. Kosgodage et al. found that CBD-treated cells, alone and with TMZ, increased extracellular vesicles (EV) containing anti-oncogenic miR-126 [63]. There were also reduced levels of pro-oncogenic miR-21 and prohibitin, which are responsible for chemo-resistant functions and mitochondria protective properties.

In pre-clinical GBM mouse models, oral administration of a Sativex-like combination of Δ^9^-THC and CBD, at a 1:1 ratio with TMZ, decreased tumor growth and increased survival [62,74]. These findings have led to two phase I/II clinical trials [77,78]. Preliminary results are only available for one study and are promising (NCT01812603) [79]. Patients with GBM were either treated with the Sativex, CBD:Δ^9^-THC (1:1), oro-mucosal spray with dose-intense TMZ, or placebo, and the first part of the study showed no Grade 3 or 4 toxicities. In the second part of this study, the same drug combination increased median survival compared to a placebo group with increased one-year survival of 83% and 56%, respectively. The most common adverse effects reported of the treatment were dizziness and nausea. Resistance to TMZ treatment may be reduced by using CBD: Δ^9^-THC combinations. When the full report is published, we are hopeful that the authors will discuss the safety and efficacy in more detail and help to determine which adverse effects can be attributed to Sativex versus TMZ.

There are also a few case studies that described the use of CBD in patients with high-grade gliomas [80,81]. Two patients were treated with procarbazine, lomustine, and vincristine along with CBD (one patient at 100–200 mg/day orally and the other at 300–450 mg/day orally) for about two years [80]. Both patients did not have any disease progression for two years after treatment. Adverse effects of the treatment included rash, moderate nausea, vomiting, and fatigue, without any lymphopenia, thrombocytopenia, hepatic toxicity, or neurotoxicity. In a case series describing nine patients with grade IV GBM, mean survival with the combination of surgery, radio- and chemo-therapy, and CBD (200–400 mg/day) was prolonged to 22.3 months, and two patients had no signs of disease progression for three or more years [81].

Taken together, the published results indicate that CBD alone, or in combination with Δ^9^-THC, TMZ, or γ-irradiation, show great promise in the treatment of glioma. Furthermore, the adverse effects of CBD alone, or together with Δ^9^-THC, appear to be relatively benign.

### 3.2. Breast Cancer

Breast cancer is the number one leading cause of new cancer cases and the second leading cause of cancer deaths of women in the United States [82]. CBD’s effects on breast cancer have been studied since 2006; research in the field has undergone recent expansion (Table S2). Various breast cancer cell lines have been used to demonstrate a dose-dependent response to CBD, including estrogen-receptor (ER)-positive cells (MCF-7, ZR-75-1, T47D), ER-negative cells (MDA-MB-231, MDA-MB-468, and SK-BR3), and triple-negative breast cancer (TNBC) cells (SUM159, 4T1up, MVT-1, and SCP2) [67,83,84,85,86,87,88]. As low as 1 to 5 µM of CBD induced significant cell death in MDA-MB-231 after 24 h [89]. CBD’s IC_50_ values for most cell lines are consistently low, indicating that breast cancer cell lines are generally sensitive to CBD’s anti-proliferative effects without a significant effect on non-transformed breast epithelial cells [87].

CBD exerts its anti-proliferative effects on breast cancer cells through a variety of mechanisms, including apoptosis, autophagy, and cell cycle arrest [67,83,87]. Ligresti et al. reported that CBD-treated MDA-MB-231 cells induced an apoptotic effect involving caspase-3, whereas CBD exerted its effects on MCF-7 through cell cycle arrest at the G_1_/S checkpoint [67]. That being said, cell cycle arrest at the G_1_/S checkpoint has been more recently demonstrated in MDA-MB-231 and 4T1 cells after CBD treatment [90]. While the activation of CB_2_ and TRPV1 receptors were seen in MDA-MB-231 cells, the effect was partial. More recent studies have found the anti-proliferative effects of CBD on breast cancer cells to be independent of the endocannabinoid receptors [87]. CBD has been consistently shown to generate ROS, which in turn inhibit proliferation and induce cell death [63,67,87,88,89]. CBD exerts its pro-apoptotic effects by downregulating mTOR, AKT, 4EBP1, and cyclin D while upregulating the expression of PPARγ and its nuclear localization [83,87]. Shrivastava et al. showed that inhibition of the AKT/mTOR signaling pathway and induction of ER stress also induced autophagy alongside apoptosis [87]. At increased CBD concentrations, or when autophagy was inhibited, the levels of apoptosis increased. They further showed that CBD may coordinate apoptosis and autophagy through the translocation and cleavage of Beclin-1.

CBD has also been shown to inhibit migration, invasion, and metastasis in aggressive breast cancer in vivo and in vitro [67,84,88,90]. McAllister et al. observed downregulated Id-1 protein by ERK and ROS in CBD-treated MDA-MB-231 and MDA-MB-436 tumors. This downregulation correlated with a decrease in tumor invasion and metastases [86,90]. Id-1 expression was also found to be downregulated in lung metastatic foci. Consistent with these observations, CBD failed to inhibit lung metastasis in Id-1 overexpressed breast cancer cells [88]. Interestingly, this same study showed that at a lower concentration (1.5 µM), which produced ROS and inhibited the expression of Id-1 in MDA-MB-231 cells, CBD did not induce autophagy or apoptosis [88]. More recently, CBD was shown to inhibit the proliferative, migratory, and invasive nature of TNBC cells by suppressing the activation of the EGF/ EGFR pathway and its downstream targets (AKT and NF-κB) [84]. MMP, phalloidin, and actin stress fibers are important in tumor invasion and were also suppressed by CBD. These results, as they pertain to EGF/EGFR pathway and the MMP, phalloidin, and actin stress fibers, were also confirmed in vivo. Primary tumor size has been shown to decrease along with the number of lung metastatic foci, volume, and vascularization in CBD-treated mice [84,90]. Intriguingly, when CBD was administered three times a week, rather than daily as was done by McAllister et al., the number of metastases were reduced and mice survived longer, but the primary tumor was not reduced [88,90]. The decreased angiogenesis and invasion were found to be due to a change in the tumor microenvironment, for example, a marked decrease in CCL3, GM-CSF, and MIP-2, which resulted in the inhibition of TAMs recruitment (Figure 4A) [84]. Finally, another study described a synthetic cannabinoid analog, O-1663, which was shown to be more potent than both CBD and Δ^9^-THC, and similarly induced cell death and autophagy [88]. O-1663 also inhibited breast cancer aggressiveness in vitro and *in vivo*. It significantly increased the survival in advanced breast cancer metastasis, inhibited the formation of metastatic foci ≥2 mm, and induced regression of established metastatic foci, all with no pronounced toxicity. Altogether, the evidence suggests that there are multiple mechanisms by which CBD impedes tumor migration.

Kosgodage et al. showed that breast cancer cells treated with CBD had increased sensitization to cisplatin. CBD significantly decreased the release of exosomes and microvesicles (EMV) (at 100–200 nm), which typically aid the spread of tumors and cause chemo-resistance [89]. However, in these same MDA-MB-231 cells, there was an increase in the release of the larger EMVs (201–500 nm). These cells displayed a concentration-dependent increase in ROS, proton leakage, mitochondrial respiration, and ATP levels. The authors attributed these effects to either a higher sensitivity or a sign of pseudo-apoptotic responses occurring, where the apoptotic factors such as ROS are still at a lower level resulting in the conversion of apoptosomes into EMVs. CBD inhibited paclitaxel-induced neurotoxicity through a 5-HT1A receptor system without conditioned reward or cognitive impairment [85]. It also decreased the viability of both 4T1 and MDA-MB-231 cells. Thus, CBD may be a viable adjunctive treatment for breast cancers as it can sensitize cells, allowing for potentially lower doses of such toxic chemicals to be prescribed.

Taken together, CBD has been consistently shown to be efficacious in many breast cancer cells and mouse models when it comes to its anti-proliferative and pro-apoptotic effects, while the mechanisms of these effects may vary. At this point, there is an urgent need for clinical trials looking at the anti-tumor effect of CBD for breast cancers, as this seems to be the next logical step in the progression of developing CBD as a treatment alternative for breast cancers.

### 3.3. Lung Cancer

Based on epidemiological studies by the American Cancer Society, lung cancer is the second most common cancer in both males and females [82]. Lung cancers are classified as small cell lung cancer (SCLC, 13%) and non-small cell lung cancers (NSCLC, 84%), which can be further subdivided into adenocarcinoma, squamous cell carcinoma, and large cell carcinoma.

Ramer and colleagues have published many studies on the effects of CBD on lung cancers (Table S3) [91,92,93,94]. They consistently used the WST-1 assay to assess the viability of lung cancers. CBD decreased the viability of two NSCLC cell lines, A549 (a lung adenocarcinoma cell line) and H460 (a large cell lung carcinoma cell line), with IC_50_ values of 3.47 µM and 2.80 µM, respectively [94]. There was a 29% and 63% reduction in A549 invasion after incubation with 0.001 µM or 0.1 µM CBD, respectively, for 72 h [92]. There was no significant cell death detected in A549 cells after treatment with 0.001 µM or 0.1 µM CBD. Various lung cancer cell lines (e.g., A549, H358, and H460) have been shown to express CB_1_, CB_2_, and TRPV1, which the anti-invasive function of CBD partly relies on [91,92,93]. CBD also significantly reduced tumor size and lung metastatic nodules (from an average of 6 nodules to only 1 nodule) in an A549 xenograft tumor model [92,93].

One mechanism of the pro-apoptotic effect of CBD is through the activation of COX-2, a pathway for endocannabinoid degradation, and PPAR-γ [94]. CBD treatment, at 3 µM in A549, H460, and primary lung tumor cells from a patient with brain metastasis, resulted in the upregulation of COX-2 and PPAR-γ both mRNA and protein. These observations were also confirmed in vivo. COX-2-derived products (PGE_2_, PGD_2,_ and 15d-PGJ_2_) were also elevated in CBD-treated lung cancer cells. By suppressing COX-2 and PPAR-γ activity with antagonists or siRNA, CBD’s pro-apoptotic and cytotoxic effects were severely attenuated. Consistently, in a lung tumor mouse model, PPAR-γ inhibition by GW9662 reversed the tumor-suppressive effects of CBD.

While Ramer et al. discussed plasminogen activator inhibitor-1's (PAI-1) pro- vs. anti-tumorigenic actions, they provided evidence supporting the former [92]. At 1 µM CBD, there was a decrease in PAI-1 mRNA and protein in A549, H358, and H460. This was confirmed in vivo using the A549 mouse model with 5 mg/kg CBD three times a week. In vitro, CBD’s anti-invasive property was reduced by siRNA knockdown of PAI-1 and was increased with the treatment of a recombinant PAI-1. The CBD-mediated decrease in PAI-1 is due, in part, to the activation of CB_1_, CB_2_, and TRPV1, as their antagonists reversed the effect. Therefore, CBD works as an agonist of CB_1_, CB_2_, and TRPV1 in lung cancers.

**Figure 5:**
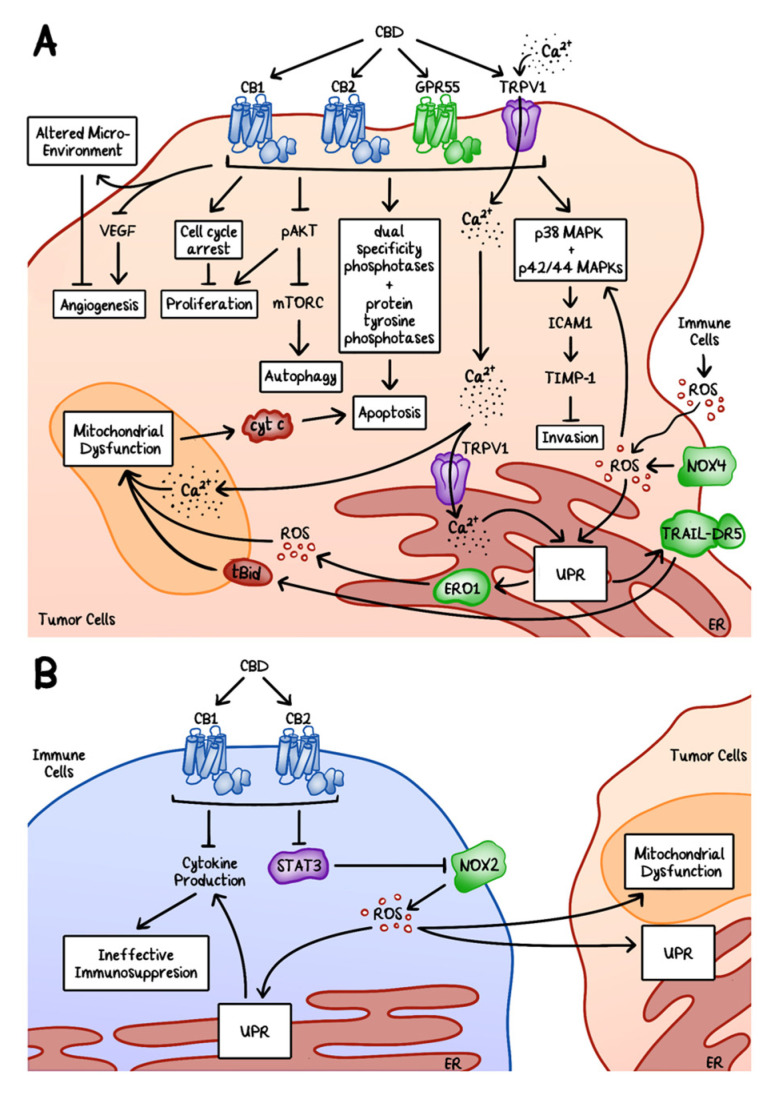
CBD’s effects on cancer cells and infiltrating immune cells. (**A**) Through its interactions with the CB_1_, CB_2_, and TRPV1 receptors, CBD induces cell cycle arrest and apoptosis in cancer cells. (**B**) CBD also binds the CB_1_ and CB_2_ receptors on the infiltrating inflammatory cells and disrupts the pro-tumorigenic cytokine production, thus leading to ineffective immunosuppression and promoting tumor cell death. ROS production by phagocytic cells disrupts the ER and mitochondrial homeostasis in tumor cells leading to apoptosis. UPR: unfolded protein response.

Tissue inhibitor of MMPs (TIMPs) were evaluated and are related to the anti-invasive effect of CBD and were found to be induced by CBD in a time- and concentration-dependent manner [93]. CBD-mediated upregulation of TIMP-1 was attributed to the activation of CB_1_, CB_2_, and TRPV1. CBD also activated p38 MAPK and p42/44 MAPK, two downstream targets of TRPV1. To connect CB_1_, CB_2_, and TRPV1 to the activation of MAPK and TIMP-1, Ramer et al. investigated the expression and function of intercellular adhesion molecule-1 (ICAM-1), a transmembrane glycoprotein involved in tumor metastasis [91] (Figure 5A). Time- and concentration-dependent increase of ICAM-1 was observed in CBD-treated A549, H358, H460, and cells from a patient with brain metastatic NSCLC. An increase in the expression of TIMP-1 mRNA was also observed, but it occurred after an increase of ICAM-1 mRNA. The expression of ICAM-1 was dependent on the activation of p42/44 MAPK and p38 MAPK. In the in vivo A549 model displaying CBD’s anti-invasive properties, both ICAM-1 and TIMP-1 were also upregulated. Inactivation of ICAM-1 using a neutralizing antibody and siRNA led to a decrease in TIMP-1 activation as well as a reduction in CBD’s anti-invasive properties. These data suggest that the MAPKs activate ICAM-1, which then stimulates the function of TIMP-1 that, in turn, suppresses tumor invasion.

In a separate study, Haustein et al. investigated CBD-induced ICAM-1 expression on lymphokine-activated kill (LAK) cell-mediated cytotoxicity [95]. Treatment with 3 µM CBD induced ICAM-1 expression and LAK cell-mediated tumor cell lysis in A549 and H460, along with metastatic cells from a patient with NSCLC. The increased susceptibility to adhesion and lysis by LAK in CBD-treated cells was reversed using a neutralizing ICAM-1 antibody. This cell lysis effect was reversed with the usage of ICAM-1 siRNA, along with CB_1_, CB_2_, and TRPV1 antagonists. Lymphocyte function association antigen (LFA-1) reversed CBD-induced killing effects on LAK cells, suggesting that it works as a counter-receptor to ICAM-1 [95]. Finally, CBD did not induce LAK cell-mediated lysis and upregulation of ICAM-1 of non-tumor bronchial epithelial cells, suggesting this effect is specific to cancer cells.

Taken together, these studies suggest that through CB_1_, CB_2_, and TRPV1 receptors, CBD activates p38 MAPK and p42/44 MAPK, which first induce ICAM-1 and then TIMP-1. The upregulation of ICAM-1 and TIMP-1 then attenuates the invasion of lung cancers (Figure 5A).

At present, there are no published results on a clinical trial using CBD to treat lung cancer patients. However, in a recent case report, an 81-year-old male patient attempted to self-treat his lung adenocarcinoma using CBD oil [96]. When first diagnosed with a mass 2.5 × 2.5 cm in size and multiple mediastinal masses, the patient was denied chemotherapy and radiation therapy given his age and the toxicity profile of these treatments. However, a year later, computed tomography (CT) scan showed that the tumor and mediastinal lymph nodes began to regress. During that period, the primary factor that was changed was that he began taking 2% CBD oil. Adverse effects included slight nausea and sickening taste.

### 3.4. Colorectal Cancer

In the US, colorectal cancer (CRC) is the third leading cause of cancer deaths in both males and females [82]. Studies using two CRC cell lines, Caco-2 and DLD-1, as well as healthy and cancerous tissues from nine CRC patients, suggest that endocannabinoid production is significantly increased in precancerous adenomatous polyps and, to a lesser extent, cancerous colon tissue [97]. Normal human colorectal tissue does express both CB_1_ and CB_2_, along with AEA, 2-AG, and endocannabinoid-metabolizing enzymes such as FAAH. Transformed adenomatous polyps have increased levels of 2-AG compared to normal colorectal tissues. While DLD-1 cells express both CB_1_ and CB_2_, Caco-2 cells only express CB_1_. Depending on the stage of the cancer, endocannabinoids can either inhibit or promote the growth of CRC. Thus, based on the stage of the cancer, both activators and inhibitors of the endocannabinoid system may be useful in combating CRC.

CBD’s effects on CRC are summarized in Table S4. The dose-dependent killing of CRC cells by CBD has been demonstrated by many studies, however, the IC_50_ values of SW480 have been reported to be as low as 5.95 µM and as high as 16.5 µM over a 48 h period [98,99,100]. This dose-dependent killing response is specific to CRC cells and not normal human colorectal cells [101]. The IC_50_ value for CaCo-2 was reported as 7.5 ± 1.3 µM [67]. Under the physiologic oxygen conditions in the colon, estimated around 5%, Caco-2 were even more sensitive to CBD, showing a decline in proliferation at 0.5 µM compared to 1 µM under atmospheric oxygen (~20%) [102]. The same study found that under physiologic oxygen conditions, the anti-proliferative effects of CBD are likely due to its ability to induce mitochondrial ROS. Apoptosis has been described as the main pathway of cell death by CBD in CRC [98,101,103].

Sreevalsan et al. used SW480 cells with 15 µM of CBD to show that the apoptosis was phosphatase- and endocannabinoid-dependent [98]. After 24 h, CBD induced the expression of various dual-specificity phosphatases and protein tyrosine phosphatases, including DUSP1, DUSP10, serum ACPP, cellular ACPP, and PTPN6. Consistent with the hypothesis, apoptosis was reduced with the use of a phosphatase inhibitor, sodium orthovanadate (SOV). Knocking down CB_1_ and CB_2_ also inhibited apoptosis. Together, these studies indicate that the apoptotic effect of CBD in CRC is through the endocannabinoid system and the activation of its downstream targets, including various phosphatases.

CBD has been shown to induce Noxa-mediated apoptosis through the generation of ROS and excessive ER stress [101]. In HCT116 and DLD-1 cells, CBD treatment induced ROS overproduction, especially mitochondrial superoxide anion, and this was linked to Noxa activation. Jeong et al. also found that Noxa-activated apoptosis was dependent on excessive ER stress from ATF3 and ATF4 [101]. These proteins bind the Noxa promoter and stimulate its expression. Similarly, in vivo, CBD-treated CRC tumors also resulted in a significant decrease in tumor size and induction of apoptosis by Noxa.

Using HCT115 and Caco-2 cells, Aviello et al. found that 10 µM of CBD exerts anti-proliferative effects through multiple mechanisms [104]. CBD may act through indirect activation of the receptors by increasing endocannabinoids, specifically 2-AG, in CRC cell lines. In vivo, CBD at 1 mg/kg significantly reduced azoxymethane-induced aberrant crypt foci, polyps, tumors, and the percentage of mice bearing polyps. CBD’s antitumor mechanism was determined to be through the downregulation of the PI3K/AKT pathway and the upregulation of Caspase-3.

A few studies also investigated CBD as an adjunctive to chemotherapy for CRC [101,103]. CRC is often treated surgically in conjunction with the combination of 5-fluorouracil, leucovorin, and oxaliplatin (FOLFOX). Seeking to overcome the potential resistance to FOLFOX, Jeong et al. treated oxaliplatin resistant DLD-1 and colo205 cells with oxaliplatin and CBD (4 µM) and found that CBD was able to enhance oxaliplatin-mediated autophagy through decreased phosphorylation of NOS3, which is involved in the production of nitric oxide (NO) and plays a role in oxaliplatin resistance [100]. The combination of oxaliplatin and CBD caused mitochondrial dysfunction (decreased oxygen consumption rate, mitochondrial membrane potential, mitochondrial complex I activity, and the number of mitochondria) through reduced SOD2 expression. These results were confirmed in vivo as well.

An alternative targeted therapy for CRC cancer, TNF-related apoptosis-inducing ligand (TRAIL), has also displayed resistance that can be overcome with the addition of CBD (4 µM) in HCT116, HT29, and DLD-1 cells [103]. CBD and TRAIL increased apoptosis through the activation of ER stress-related genes, including PERK, CHOP, and DR5. In vivo, TRAIL with CBD showed a significant decrease in tumor growth and an increased number of apoptotic cells. Altogether, these FOLFOX and TRAIL therapy studies suggest that CBD may be considered as a therapeutic option for CRC or, perhaps, as an adjunctive treatment to work synergistically with conventional chemotherapies. Currently, there are no clinical trials related to CBD in CRC, however, these findings related to the synergistic effects of CBD with chemotherapies are very promising and make a good case for a clinical trial in the future.

### 3.5. Leukemia/Lymphoma

Our understanding of CBD’s effects on leukemia and lymphoma has expanded in recent years (Table S5). EL-4 and Jurkat cell lines are the commonly used models for lymphoma and leukemia, respectively. CBD induced a dose- and time-dependent killing effect on these leukemia and lymphoma cell lines, whereas peripheral blood monomolecular cells were more resistant to CBD [105,106,107,108,109].

McKallip et al. [106] found that in both EL-4 and Jurkat cells, CBD’s anti-proliferative effects were mediated through CB_2_, but independent of CB_1_ and TRPV1 [106]. However in a separate study Olivas-Aguirre et al. showed CBD’s effects to be independent of the endocannabinoid receptors and plasma membrane Ca^2+^ channels in Jurkat cells [110]. These conflicting results need to be resolved by future studies. Despite this, the majority of research on leukemia/lymphomas confirmed apoptosis as the mechanism by which CBD-mediated cell death occurs, either alone or in combination with other treatment modalities, including γ-irradiation, Δ^9^-THC, vincristine, and cytarbine [105,106,107,110]. One study also demonstrated that CBD decreased tumor burden and induced apoptosis in vivo [106]. Kalenderoglou et al. found that CBD can induce cell cycle arrest in Jurkat cells, with increased cells in G1 phase [108]. CBD treatment also resulted in changes to cell morphology, including decreased size of cells, extensive vacuolation, swollen mitochondria, disassembled ER and Golgi, and plasma membrane blebbing [108,110].

Similar to the results of other cancers as discussed above, CBD also induced ROS in leukemia and lymphoma [106,110,111]. Treating Jurkat and MOLT-4, another leukemia cell line, with ≥2.5 µM CBD for 24 h induced increased ROS levels [106]. Treating cells together with ROS scavengers, α-tocopherol and NAC, reduced CBD’s killing effects. CBD exposure also increased NOX4 and p22^phox^ while inhibiting NOX4 and p22^phox^ decreased ROS levels and inhibited CBD-induced cell toxicity. Consistent with these observations, ROS levels were significantly increased after only two hours of CBD treatment in EL-4 cells, with a concomitant decrease in cellular thiols [111].

Kalenderoglou et al. explored CBD’s effects on the mTOR pathway in Jurkat cells [108]. They found that CBD reduced the phosphorylation of AKT and ribosomal protein S6. They also tested CBD’s effects with different nutrient and oxygen conditions and found that CBD’s anti-proliferative effects alone or together with doxorubicin were greater with 1% serum than 5% serum. Olivas-Aguirre et al. found that when Jurkat cells were treated with lower concentrations of CBD, proliferation still occurred (at 1 µM CBD) and autophagy was increased at 10 µM CBD [110]. However, at higher concentrations (30 µM), the intrinsic apoptotic pathway was activated, resulting in cytochrome c release and Ca^2+^ overload within the mitochondria. In Burkitt lymphoma cell lines, Jiyoye and Mutu I, AF1q stimulated cell proliferation and reduced ICAM-1 expression, through which cells became resistant to chemotherapies [104]. After exposure to CBD for 24 h, the chemo-resistant effect was dramatically attenuated.

### 3.6. Prostate Cancer

Prostate cancer is the most common cancer and the second most common cause of cancer-related deaths in men [82]. The detailed summary of studies describing CBD’s effects on prostate cancer can be found in Table S6. The prostate cancer cell lines used in those studies can be divided into androgen receptor (AR)-positive (LNCaP and 22RV1) and AR-negative (DU-145 and PC-3). CBD can inhibit the expression of the androgen receptor in AR-positive cell lines [112]. Regarding the endocannabinoid receptors, depending on the specific cancer cell type, either CB_1_, or CB_2_, or both, are upregulated in prostate cancer cells relative to normal prostate cells [112,113]. Specifically, 22RV1 only expresses CB_1_ while DU-145 only expresses CB_2_. Though CB_1_ and CB_2_ can be found in both LNCaP and PC-3, their levels are much more prominent in PC-3. TRPV1 is expressed in all four prostate cancer cell lines, with the highest expression found in DU-145 cells.

CBD induced anti-proliferative effects and apoptosis-mediated cell death (via the intrinsic pathway) in prostate cancer cells, which may be dependent on CB_2_, but not CB_1_, and the transient receptor potential cation channel subfamily M member 8 (TRPM8) receptor in LNCaP cells [112,113]. Additionally, treatment with CBD was shown to downregulate the expression prostate-specific antigen (PSA), vascular endothelial growth factor (VEGF), and pro-inflammatory cytokines [113]. CBD treatment resulted in cell cycle arrest at G0/G1 transition in LNCaP and PC3 cells and G1/S transition in DU-145 cells.

Similar to the CRCs, Sreevalsan et al. found that dual-specificity phosphatases and protein tyrosine phosphatases were also induced by CBD in LNCaP cells [98]. Inhibition of the phosphatases with the phosphatase inhibitor, SOV, decreased PARP cleavage. Additionally, CBD enhanced the phosphorylation of p38 MAPK. Most recently, Kosgodage et al. found that in PC3, CBD treatment (1 µM and 5 µM) reduced the release of EMV [89,114]. CBD was also shown to reduce mitochondrial-associated proteins, prohibitin, and STAT3, which may account for the decrease of EMV.

At this point, only one study testing CBD’s effectiveness on prostate cancer has been conducted *in vivo.* More quality studies using mouse models are required before moving to the clinical trial phase.

### 3.7. Other Cancer Types:

The effects of CBD on a variety of other cancers have also been reported, however to a lesser degree (Table S7). Cervical cancer cell lines treated with CBD had time- and concentration-dependent killing effects that were shown to be mediated by apoptosis and independent of cell cycle arrest [93,115]. Treatment with CBD resulted in the upregulation of p53 and Bax, a pro-apoptotic protein, and downregulation of RBBP6 and Bcl-2, two anti-apoptotic proteins, in SiHa, HeLa, and ME-180 cells [115]. CBD also decreased the invasion of HeLa and C33A, which was dependent on CB_1_, CB_2_, and TRPV1. Ramer et al. also found this anti-invasive property of CBD to be associated with the upregulation of p38 MAPK and p42/44 MAPK, along with their downstream target, TIMP-1, which is similar to lung cancers as discussed above (Figure 5A).

CBD (1 µM and 5 µM) also decreased the cell viability of a hepatocellular carcinoma cell line, Hep G2, in a dose-dependent manner after 24 h [89]. Similar to the breast and prostate cell lines, MDA-MB-231 and PC3, respectively, CBD-treated Hep G2 cells reduced the release of EMV and the expression of CD63, prohibitin, and STAT3. Additionally, treating Hep G2 cells with CBD sensitized them to cisplatin. Neumann-Raizel et al. used the mouse hepatocellular carcinoma cell line, BNL1 ME, which expresses functional TRPV2 channels, to demonstrate the effects of CBD in conjunction with doxorubicin [116]. CBD (10 µM) was shown to activate TRPV2 and inhibit the P-glycoprotein ATPase transporter, allowing for increased entry and accumulation of doxorubicin into the cell since it is transported across the cytoplasmic membrane through TRPV2 and pumped out of the cell using the P-glycoprotein ATPase transporter. These effects were likely responsible for CBD’s ability to decrease the dose of doxorubicin required to reduce cell viability and proliferation.

Regarding thyroid cancers, CBD induced an anti-proliferative effect in KiMol through the activation of apoptosis and cell cycle arrest [67]. KiMol was shown to contain increased levels of CB_1_, CB_2_, and TRPV1, but inhibitors of CB_1_, CB_2_, and TRPV1 only slightly decreased the anti-proliferative effects of CBD. CBD (5 mg/kg twice per week) produced anti-tumor effects in a mouse thyroid tumor model as well.

Taha et al. studied patients with stage IV non-small cell lung cancer, clear cell renal cell carcinoma, and advanced melanoma treated with nivolumab immunotherapy (anti-PD-1 agents) and patients who had additionally used cannabis, including CBD and Δ^9^-THC [117]. They showed a decreased response rate to treatment in groups using cannabis with nivolumab, whereas patients not using cannabis were 3.17 times more likely to respond to treatment with nivolumab. However, cannabis use resulted in no significant difference in overall survival and progression-free survival. This group suggested that there may be a possible negative interaction between cannabis and immunotherapy.

CBD decreased cell proliferation and colony formation in a concentration-dependent manner in gastric cancer cells without affecting normal gastric cells [67,118,119]. The gastric adenocarcinoma cell line, AGS, has abundant expression of TRPV1 without the detection of CB_1_ or CB_2_ [67]. Zhang et al. found that CBD induced cell cycle arrest by inhibiting the expression of CDK2 and cyclin E in SGC-7901, another gastric cancer cell line [119]. In addition, CBD increased the expression of ATM and p21, while decreasing that of p53. CBD’s anti-proliferative effects in SGC-7901 were also attributed to mitochondrial-dependent apoptosis, as it increased the activity of Caspase-3 and Caspase-9, the release of cytochrome c, and the expression of Apaf-1, Bad, and Bax proteins and decreased the expression of Bcl-2. CBD-induced cell cycle arrest and apoptosis were associated with increased ROS levels. In multiple gastric cancer cell lines, Jeong et al. showed that CBD caused apoptosis by inducing ER stress, which then upregulated the second mitochondria-derived activator of caspase (Smac) [118]. Smac upregulation resulted in downregulation of X-linked inhibitor of apoptosis (XIAP) through ubiquitination/proteasome activation. CBD was also shown to induce mitochondrial dysfunction (Figure 5A), as shown by CBD-driven decreases in oxygen consumption rate, ATP production, mitochondrial membrane potential, and NADH dehydrogenase ubiquinone 1α sub-complex subunit 9. *In vivo*, mice injected with MKN45, another gastric cancer cell line, showed slower tumor growth and smaller tumor size with CBD treatment (20 mg/kg) three times a week. Like the in vitro studies, CBD promoted apoptosis and decreased the expression of XIAP in the tumors.

Melanoma cancer cell lines (B16 and A375) express the endocannabinoid receptors, CB_1_, and CB_2_ [120]. Previous studies have also shown that activation of these receptors with Δ^9^-THC decreased melanoma growth, proliferation, angiogenesis, and metastasis in vivo [120]. While Δ^9^-THC looks promising as a treatment modality of melanoma, there has been little research on the effects of CBD on melanoma. A recent study by Simmerman et al. tested CBD in a murine melanoma model (B16F10) [121]. They set up three groups of mice: control (ethanol- and PBS-treated), cisplatin-treated (5 mg/kg intraperitoneal once per week), and CBD-treated (5 mg/kg intraperitoneal twice a week). Survival time was significantly increased, and tumor size was significantly decreased in CBD-treated mice compared to control mice, but to a lesser effect when compared to that of cisplatin-treated mice. Quality of life was subjectively described, and CBD-treated mice were found to have a better quality of life, improved movement, and less hostile interaction/fighting compared to both controls and cisplatin-treated mice. This study did not include a group of CBD and cisplatin combination treatment. More research is required to understand the effects of CBD on human melanoma cells.

Pancreatic cancers, especially pancreatic ductal adenocarcinoma (PDAC), have seen few improvements in treatment and survival. Ferro et al. used PDAC cancer cell lines, including ASPC1, HPAFII, BXPC3, and PANC1, as well as the KRAS^Wt/G12D^/TP53^WT/R172H^/Pdx1-Cre^+/+^ (KPC) mice as models of PDAC to demonstrate GPR55 accumulating in PDAC tissue, and that its disruption resulted in improved survival and reduced proliferation both in vivo and in vitro [122]. This mainly occurred via cell cycle arrest at the G1/S transition by reducing the expression of cyclins, without increasing apoptosis. Additionally, they found downstream MAPK/ERK signaling to be inhibited in cells depleted of GPR55. In vivo, treatment of KPC mice with CBD (100 mg/kg) increased survival similar to gemcitabine (GEM) (100 mg/kg), and when CBD and GEM were used together survival was increased about three-fold compared to the control. With this combination, cell proliferation was also reduced. CBD was also able to counteract the increased ERK activation by GEM, a proposed mechanism of acquired GEM resistance.

## 4. Summary and Conclusions

As evidenced by the large volume of literature reviewed above, CBD has demonstrated robust anti-proliferative and pro-apoptotic effects on a wide variety of cancer types both in cultured cancer cell lines and in mouse tumor models. In comparison, CBD generally has milder effects on normal cells from the same tissue/organ. The anti-tumor mechanisms vary based on tumor types, ranging from cell cycle arrest to autophagy, to cell death, or in combination. In addition, CBD can also inhibit tumor migration, invasion, and neo-vascularization (Figure 5A), suggesting that CBD not only acts on tumor cells but can also affect the tumor microenvironment, for example by modulating infiltrating mesenchymal cells and immune cells. The dependency of CBD on the endocannabinoid receptors, CB_1_ and CB_2_, or the TRPV family of calcium channels, also varies, suggesting that CBD may have multiple cellular targets and/or different cellular targets in different tumors (Table 1). Mechanistically, CBD seems to disrupt the cellular redox homeostasis and induce a drastic increase of ROS and ER stress, which could then exert the cell cycle arrest, autophagy, and cell death effects (Figure 5A). For future studies, it is crucial to elucidate the interplays among different signaling transduction pathways, such as ROS, ER stress, and inflammation, in order to better understand how CBD treatment disrupts cellular homeostasis in both tumor cells as well as infiltrating cells, leading to cancer cell death and inhibition of tumor migration, invasion, metastasis, and angiogenesis. The final step of developing CBD as an oncology drug is through extensive and well-designed clinical trials, which are urgently needed.

### 4.1. The Cellular Targets of CBD

Though the affinity of CBD to CB_1_ and CB_2_ is considered relatively low, both CB_1_ and CB_2_ could still be the targets of CBD in certain cancer cells and in infiltrating cells in the tumor microenvironment. Other identified cellular targets of CBD include TRPV1, TRPV2, GPR55, and possibly other GPCRs or non-GPCRs. As summarized in Table 1, these cellular targets can vary depending on cancer types. For example, CBD’s effects in glioma are dependent on TRPV2, but not on CB_1_, CB_2_, and TRPV1 [58,66,67,69,72,106]. On the other hand, CBD’s effects in lung, colorectal, prostate, and cervical cancers are largely dependent on some combination of CB_1_, CB_2_, and TRPV1 [91,92,93,95,98,113]. The simple presence of these receptors on the surface of cancer cells is not necessarily a good predictor of CBD sensitivity. For example, CB_1_, CB_2_, and TRPV1 are highly expressed on the cell surface of the thyroid cancer cell line, SkiMol; however, inhibition of these receptors only mildly affected the anti-proliferative effect of CBD in SkiMol [67].

### 4.2. CBD Induces Intracellular ROS and ER Stress and Enhances the Immune Response

Though the cellular response to CBD treatment can be quite complex, certain themes have emerged to explain its anti-tumor effects. One shared feature of CBD-treated cancer cells is the drastic elevation of ROS (Table 1), likely caused by the disruption of intracellular calcium homeostasis and/or mitochondrial functions. ER stress and ROS production are highly related and tightly regulated via ERO1 activity [123] (Figure 5A). Each pathway can activate the other, but they ultimately culminate with the activation of mitochondria-mediated cell death due to increased intracellular calcium. The upstream regulation of ROS- and ER stress-induced apoptosis is largely unknown. One of the possible mechanisms is through the TRPV channels. For example, Wang et al. demonstrated that treatment of ovarian cancer cells with the TRPV1 antagonist, DWP05195, increased ROS production via NOX upregulation; increased ROS upregulated CHOP activity leading to ER stress-mediated apoptosis. Interestingly, the TRPV1 antagonist did not drastically change calcium levels [124]. This suggests another possible mechanism of intracellular calcium regulation—the NOX enzymes. Calcium release from the ER was shown to activate NOX, leading to ROS production in endothelial cells [125]. Whether CBD-induced ER stress and ROS generation are mediated through activation of the CB_1_, CB_2_, TRPV1 or other channels needs further investigation. CBD may regulate intracellular calcium via transmembrane channels or ER release leading to apoptosis.

The endocannabinoid receptors, CB_1_ and CB_2_, are highly expressed on inflammatory cells, including B cells, NK cells, monocytes, T cells, and neutrophils. Furthermore, CB_2_ is differentially expressed as B cells and macrophages become activated. Studies regarding the immune-modulatory role of the endocannabinoid system have shown that CB_2_ activation inhibits the production of TNF-α, IL-6, and IL-8 in monocytes and macrophages [126]. Not surprisingly, CBD reduced TNF-α production in macrophages after LPS stimulation. In addition, CBD also decreased the secretion of IL-1β and TNF-α from activated lymphocytes and monocytes in the peripheral blood.

Secretion of cytokines is also largely mediated by the production of ROS, a major source being the NOX2-expressing immune cells. MSDCs produce ROS in many cancer types through increased expression of NOX2 which is regulated by STAT3 [127]. CBD was shown to reduce STAT3 levels in colorectal cancers, prostate cancers, hepatocellular carcinoma, breast cancers, leukemia and lymphomas [89,101,106]. MDSCs lacking NOX2 were unable to prevent T cell proliferation and IFNγ production [128,129]. Thus, STAT3 inhibition via CBD increases the Th1 immune response and is a major source of ROS production, leading to tumor cell death. Whether STAT3 downregulation in tumor-associated immune cells is mediated by the CBD agonist or inverse agonist effects on CB_2_ receptors needs to be further investigated.

### 4.3. Safety of CBD in Humans

Most research regarding the effects of CBD on cancers has yet to reach the clinical trial phase, thus we are limited in understanding the safety profile at the doses required to inhibit tumor growth. The study of CBD and Δ^9^-THC in GBM treatment by Twelves et al. described dizziness and nausea as the most common adverse effects [79]. Outside of the realm of cancer treatment, CBD was shown to be safe without inducing changes in heart rate, blood pressure, neurologic testing, or blood tests [130]. Unlike other controlled substances, patients do not seem to develop a tolerance for CBD [131]. Drug interactions with CBD may occur as it also affects the expression of various CYP enzymes, thus caution should be taken in patients on medications metabolized in the liver [130].

### 4.4. An Urgent Need for Clinical Trials

As discussed above, there is extensive preclinical research indicating CBD as an efficacious anti-cancer agent either alone or in conjunction with other cannabinoids, chemotherapies, and radiation therapy. Even though CBD does cause mild hepatotoxicity in mice and cats, preliminary toxicity studies suggest that there may still be a therapeutic window for cancer therapy in humans [132,133,134]. Therefore, systematic clinical trials into CBD that determine its safety and efficacy in a variety of cancers are the next logical step in developing CBD as an oncology drug. This could be done with CBD alone or in combination with established therapeutic modalities.

## Figures and Tables

**Table 1:** The effects of CBD on different cancers.

				Potential Cellular Targets					Anti-Tumor Pathway		
Tumor Type	ROS	ER Stress	Inflammation	CB_1_	CB_2_	TRPV1	TRPV2	GPR55	PI3K/AKT/mTOR	MAPK	Autophagy
Glioblastoma	↑	↑	↑	X	X	X	↑		↓	↑	↑
Breast	↑	↑		X	X	X			↓		↑
Lung				↑	↑	↑				↑	
Colon	↑	↑		↑	↑	↑			↓		↑
Leukemia/lymphoma	↑	↑		X	↑	X			↓	↑	
Prostate	↑	↑	↑	X	↑					↑	
Cervical				↑	↑	↑				↑	
Gastric	↑	↑									
Pancreatic								↓		↓	

↑: increase the activity/amount; ↓: decrease the activity/amount; X: not involved.

## Supplementary Materials

The following are available online at https://www.mdpi.com/2072-6694/12/11/3203/s1, Table S1: CBD’s effect on Gliomas, Table S2: CBD’s effect on Breast Cancers, Table S3: CBD’s Effect on Lung Cancers, Table S4: CBD’s effect on Colorectal Cancers, Table S5: CBD’s effect on Leukemia and Lymphoma, Table S6: CBD’s effect on Prostate Cancers, Table S7: CBD’s Effect on Other Cancer Types.
