# Supplementary material for: Cannabidiol (CBD) as a Promising Anti-Cancer Drug
Source: Cancers (Basel). 2020 Oct 30;12(11):3203. doi: 10.3390/cancers12113203 (PMC7693730; doi:10.3390/cancers12113203)
Supplement: Supplementary file 1 [file cancers-12-03203-s001.pdf]

Supplementary Material: Cannabidiol (CBD) as a Promising Anti-cancer Drug

Emily S. Seltzer, Andrea K. Watters, Danny MacKenzie, Lauren M. Granat and Dong Zhang

Table S1. CBD's effect on Gliomas.

Paper	Glioma cell line	IC_{50} (μM)	Effect on cancer cells	Mechanism of action	Tested in cell line?	Tested in mouse model?
Jacobsson et al. (2000) [1]	C6	ND<br>*	Enhances Tamoxifen's anti-proliferative effect	N.D.	Yes	No
Massi et al. (2003) [2]	U87<br>U373	26.2 ± 2.8<br>24.1 ± 2.16<br>*	Anti-proliferative<br>Apoptosis<br>Decreases tumor size	Partially through CB_2<br>Independent of CB_1 and vanilloid receptor<br>Independent of Gi/Go-coupled receptor mechanism<br>Induces oxidative stress	Yes	Yes
Vaccani et al. (2005) [3]	U87	5.5 ± 1.1<br>(*Cell migration*)	Inhibits cell migration	Independent of cannabinoid, vanilloid receptors and TRPVs<br>Independent of Gi/o-coupled receptors	Yes	No
Massi et al. (2006) [4]	U87<br>Normal glial cell	ND<br>(*Concentration that was inhibitory:* 25 μM *vs non-inhibitory:* 10 μM)<br>*	Anti-proliferative<br>Apoptosis	Intrinsic and extrinsic caspase-dependent<br>Increases ROS and reduces glutathione	Yes	No
Ligresti et al. (2006) [5]	C6	8.5 ± 0.8<br>**	Anti-proliferative<br>Apoptosis	Independent of cell cycle arrest<br>Independent of CB_1, CB_2 and TRPV1	Yes	No
Massi et al. (2008) [6]	U87	ND<br>*	Anti-tumor growth	Enhances FAAH degradation<br>Reduces AEA and AEA-binding cannabinoid receptors<br>Decreases 5-LOX activity and levels<br>Independent of COX-2	Yes	Yes
Marcu et al. (2010) [7]	SF126<br>U251<br>U87	1.2 (1.1-1.3)<br>0.6 (0.5-1.0)<br>0.6 (0.5-0.7)<br>*	Anti-proliferative (alone and synergistically with Δ^9-THC)<br>*Synergistically with Δ^9-THC*: Anti-invasive	Induces ROS (alone and with Δ^9-THC)<br>*Synergistically with Δ^9-THC*: Downregulation of phosphorylation of p42/44 MAPK	Yes	No

			Apoptosis<br>Inhibits cell cycle	Partial dependence on CB_2 receptor		
Torres et al. (2011) [8]	U87MG<br>A172<br>SW1783<br>U372<br>T98<br>SW1088<br>LN405<br>HG19<br>HG2<br>HG14	1.49<br>1.49<br>*	Antiproliferative<br>Apoptosis<br>Reduces tumor growth<br>(Allows for lower dose of Δ^9-THC)	Independent of CB_1, CB_2, ceramide biosynthesis, and autophagy<br>Induces ROS	Yes	Yes
Nabissi et al. (2013) [9]	U87MG<br>MZC	30.2<br>33.2<br>*	Antiproliferative<br>Apoptosis (at >10 μM)<br>Potentiates cytotoxic effect of chemotherapies (BCNU, TMZ, DOXO)	Acts as a selective TRPV2 agonist, enhancing calcium influx<br>(specifically, at the pore region of the TRP channels)<br>Increases expression of TRPV2	Yes	No
Solinas et al. (2013) [10]	U87MG<br>T98G	11.16 (10.94-11.38)<br>13.41 (13.09-13.86)<br>*	Decreases cell invasion<br>Antiproliferative	*U87-MG:* inhibits of MMP-9, TIMP-1, TIMP-4, uPA, SerpinE1-PAI-1 and VEGF<br>*T98G:* inhibits MMP-9, TIMP-4, SerpinE1-PAI-1, VEGF, TGF-b1, CXCL-16 and PDGF-AA<br>Downregulates ERK and PI3K/Akt pathway<br>Inhibits HIF-1*α in U87-MG cells*	Yes	No
De La Ossa et al. (2013) [11]	U87MG	ND<br>(Used Cannabinoid-loaded MPs)	Antiproliferative<br>Decrease tumor growth and weight<br>Apoptosis	Decreases tumor vascularization	No	Yes
Scott et al. (2014) [12]	T98G<br>U87MG<br>GL261	24 h: 10 ± 2.1<br>48 h: 8.4 ± 2.9<br>72 h: 5.2 ± 1.3<br>24 h: 12 ± 3.4<br>48 h: 14 ± 7.1<br>72 h: 9.7 ± 1.3<br>24 h: 11 ± 1.0<br>48 h: 11 ± 4.2<br>72 h 10 ± 3.9<br>***	Antiproliferative<br>Reduction in tumor size<br>(Alone and combined with ionized radiation + Δ^9-THC)<br>Apoptosis (mild)<br>(cytotoxic and cytostatic)	Modulates phosphorylation of p42/44 MAPK<br>Reduces pAKT	Yes	Yes
Nabissi et al. (2015) [13]	Glioma stem-like cells<br>#1<br>#30<br>#83	19.4<br>14.6<br>19.3<br>*	Antiproliferative<br>Autophagy<br>Induces glioblastoma stem cell differentiation<br>Increases	Independent of CB_1, CB_2 and TRPV1<br>Dependent on TRPV2<br>Upregulates PTEN, reduces AKT activity<br>Increase Aml-1 transcription	Yes	No

			sensitivity to carmustine treatment via apoptosis			
Scott et al. (2015) [14]	T98G<br>U87MG	11 ± 2.7<br>17 ± 1.3<br>*	Antiproliferative	Upregulates heat shock protein which allows for an increase in ROS<br>However, heat shock proteins dampen cytotoxic effects	Yes	No
Deng et al. (2017) [15]	T98G<br><br>U251<br><br>U87MG<br><br>Mouse: PDGF-GBM<br><br>Neural progenitor cells (for CNS toxicity)	5.08 proliferation<br>6.75 viability<br>8.54 proliferation<br>9.21 viability<br>6.59 proliferation<br>5.13 viability<br><br>3.14 proliferation<br>4.98 viability<br>3.07 proliferation<br>3.19 viability<br>****	Antiproliferative<br>Cell killing<br><br>*With DNA damaging agents:*<br>Synergistic antiproliferative and cell-killing responses<br>In some lines, antagonistic responses at low concentrations	Cooperative/allosteric mechanism of action	Yes	No
Ivanov et al. (2017) [16]	U87MG<br>U118MG<br>T98G	ND	Apoptosis (CBD alone and CBD with γ-irradiation)	Upregulates active JNK1/2 and MAPK p38<br>Downregulates active phosphorylation of p42/44 MAPK and AKT1 levels<br>Upregulates TNF/TNFR1 and TRAIL/TRAIL-R2 signaling	Yes	No
López-Valero et al. (2018) [17]	U87MG<br>Glioma initiating cells:<br>GH2 GICs<br>12012 GICs	ND	*CBD + Δ^9-THC ±* TMZ*:*<br>Anti-proliferative<br>Apoptosis<br>Increases survival<br>Reduces tumor growth (only CBD + Δ^9-THC + TMZ)	N.D.	Yes	Yes
López-Valero et al. (2018) [18]	U87MG	ND<br>*	*Sativex-like combination of CBD and Δ9-THC (1:1 ratio) ± TMZ:*<br>Anti-tumor<br>Anti-proliferative<br>Apoptosis<br>Autophagy<br>Reduces Tumor	N.D.	Yes	Yes

			Size<br>Total tumor regression (>50%)<br>Increases survival			
Aparicio-Blanco et al. (2019) [19]	U373MG	29.1<br>292.6 (20 nm sized LNC 48h)<br>129.1 (20 nm sized LNC 96h)<br>615.4 (50 nm sized LNC 48h)<br>375.4 (50 nm sized LNC 96h)<br>158.6 (40 nm sized LNC - CBD functionalized 48 h)<br>513.2 (60 nm sized LNC - CBD functionalized 48 h)<br>*	Anti-proliferative	N.D.	Yes	No
Kosgodage et al. (2019) [20]	LN18 (chemo-resistant)<br>LN229 (chemo-sensitive)	ND<br>*****	*CBD ± TMZ:*<br>Anti-proliferative	Increases extracellular vesicles and within them, increases anti-oncogenic miR-126, decreased pro-oncogenic miR-21 levels<br>Reduces prohibitin (mitochondrial protective and chemo-resistant functions)	Yes	No
Ivanov et al. (2019) [21]	U87MG<br>U118MG<br>T98G	ND<br>******	*CBD + γ-irradiation + ATM inhibitor (KU60019):*<br>Apoptosis<br>Cell cycle (G2/M) arrest<br>Anti-proliferative	Increases PD-L1<br>Produces pro-inflammatory cytokines (IL1β, IL6, IL8)<br>Produces FAS-L and TRAIL<br>Upregulates DR5 and TRAIL-R2<br>Activates JNK-AP1 and *NF*-κB leading to TRAIL-mediated apoptosis	Missing into	Missing info

ND = Not determined, * MTT colorimetric assay, ** Crystal Violet vital staining/spectrophotometer, *** fluorescence from cellular reduction of resazurin to resarufin, ****WST-1, ***** Guava Via Count cell death assay, ******CellTiter-Glo Luminescent Cell Viability Assay, DOXO: doxorubicin, BCNU:

Carmustine, TMZ: temozolomide; Transient receptor potential (TRP); GBM: Glioblastoma multiforme; LNC: lipid nanocapsules (form of CBD administration). All IC50 values were converted to μM for continuity using the molar mass for CBD (314.464 g/mol). NOTE: only pure CBD results were discussed, combination with other treatment modalities were mentioned.

1. Jacobsson, S.O.P.; Rongård, E.; Stridh, M.; Tiger, G.; Fowler, C.J. Serum-dependent effects of tamoxifen and cannabinoids upon C6 glioma cell viability. *Biochem. Pharmacol.* **2000**, *60*, 1807–1813, doi:10.1016/s0006-2952(00)00492-5.
2. Massi P.; Vaccani A.; Ceruti S.; Colombo A.; Abbracchio MP.; Parolaro D., Antitumor effects of cannabidiol, a non-psychotropic cannabinoid, on human glioma cell lines. *Journal of Pharmacology and Experimental Therapeutics*. **2003,** *10*(*2*), 255–267.
3. Vaccani, A.; Massi, P.; Colombo, A.; Rubino, T.; Parolaro, D. Cannabidiol inhibits human glioma cell migration through a cannabinoid receptor-independent mechanism. *Br. J. Pharmacol.* **2005**, *144*, 1032–1036, doi:10.1038/sj.bjp.0706134.
4. Massi P.; Vaccani A.; Bianchessi S.; Costa B.; Macchi P.; Parolaro D., The non-psychoactive cannabidiol triggers caspase activation and oxidative stress in human glioma cells *Cell Mol Life Sci*. **2006**, *63*(*17*), 2057–2066.
5. Ligresti, A.; Moriello, A.S.; Starowicz, K.; Matias, I.; Pisanti, S.; De Petrocellis, L.; Laezza, C.; Portella, G.; Bifulco, M.; Di Marzo, V. Antitumor Activity of Plant Cannabinoids with Emphasis on the Effect of Cannabidiol on Human Breast Carcinoma. *J. Pharmacol. Exp. Ther.* **2006**, *318*, 1375–1387, doi:10.1124/jpet.106.105247.
6. Massi, P.; Valenti, M.; Vaccani, A.; Gasperi, V.; Perletti, G.; Marras, E.; Fezza, F.; Maccarrone, M.; Parolaro, D. 5-Lipoxygenase and anandamide hydrolase (FAAH) mediate the antitumor activity of cannabidiol, a non-psychoactive cannabinoid. *J. Neurochem.* **2008**, *104*, 1091–1100, doi:10.1111/j.1471-4159.2007.05073.x.
7. Marcu, J.P.; Christian, R.T.; Lau, D.; Zielinski, A.J.; Horowitz, M.P.; Lee, J.; Pakdel, A.; Allison, J.; Limbad, C.; Moore, D.H.; et al. Cannabidiol Enhances the Inhibitory Effects of 9-Tetrahydrocannabinol on Human Glioblastoma Cell Proliferation and Survival. *Mol. Cancer Ther.* **2010**, *9*, 180–189, doi:10.1158/1535-7163.mct-09-0407.
8. Torres, S.; Lorente, M.; Rodríguez-Fornés, F.; Hernández-Tiedra, S.; Salazar, M.; García-Taboada, E.; Barcia, J.; Guzmán, M.; Velasco, G. A Combined Preclinical Therapy of Cannabinoids and Temozolomide against Glioma. *Mol. Cancer Ther.* **2011**, *10*, 90–103, doi:10.1158/1535-7163.mct-10-0688.
9. Nabissi, M.; Morelli, M.B.; Santoni, M.; Santoni, G. Triggering of the TRPV2 channel by cannabidiol sensitizes glioblastoma cells to cytotoxic chemotherapeutic agents. *Carcinog.* **2012**, *34*, 48–57, doi:10.1093/carcin/bgs328.
10. Solinas, M.; Massi, P.; Cinquina, V.; Valenti, M.; Bolognini, D.; Gariboldi, M.; Monti, E.; Rubino, T.; Parolaro, D. Cannabidiol, a Non-Psychoactive Cannabinoid Compound, Inhibits Proliferation and Invasion in U87-MG and T98G Glioma Cells through a Multitarget Effect. *PLOS ONE* **2013**, *8*, e76918, doi:10.1371/journal.pone.0076918.
11. De La Ossa, D.H.P.; Lorente, M.; Gil-Alegre, M.E.; Torres, S.; García-Taboada, E.; Aberturas, M.D.R.; Molpeceres, J.; Velasco, G.; Torres-Suárez, A. Local Delivery of Cannabinoid-Loaded Microparticles Inhibits Tumor Growth in a Murine Xenograft Model of Glioblastoma Multiforme. *PLOS ONE* **2013**, *8*, e54795, doi:10.1371/journal.pone.0054795.
12. Scott, K.A.; Dalgleish, A.G.; Liu, W.M. The Combination of Cannabidiol and Δ9-Tetrahydrocannabinol Enhances the Anticancer Effects of Radiation in an Orthotopic Murine Glioma Model. *Mol. Cancer Ther.* **2014**, *13*, 2955–2967, doi:10.1158/1535-7163.mct-14-0402.
13. Nabissi M.; Morelli MB.; Amantini C., et al. Cannabidiol stimulates A ml-1a-dependent glial differentiation and inhibits glioma stem-like cells proliferation by inducing autophagy in a TRPV 2-dependent manner. *International journal of cancer*. **2015**, *137*(*8*), 1855–1869.
14. A Scott, K.; Dennis, J.L.; Dalgleish, A.G.; Liu, W.M. Inhibiting Heat Shock Proteins Can Potentiate the Cytotoxic Effect of Cannabidiol in Human Glioma Cells. *Anticancer. Res.* 2015, *35*.

15. Deng, L.; Ng, L.; Ozawa, T.; Stella, N. Quantitative Analyses of Synergistic Responses between Cannabidiol and DNA-Damaging Agents on the Proliferation and Viability of Glioblastoma and Neural Progenitor Cells in Culture. *J. Pharmacol. Exp. Ther.* **2017**, *360*, 215–224, doi:10.1124/jpet.116.236968.
16. Ivanov, V.N.; Wu, J.; Hei, T.K. Regulation of human glioblastoma cell death by combined treatment of cannabidiol, γ-radiation and small molecule inhibitors of cell signaling pathways. *Oncotarget* **2017**, *8*, 74068–74095, doi:10.18632/oncotarget.18240.
17. López-Valero, I.; Saiz-Ladera, C.; Torres, S.; Hernández-Tiedra, S.; García-Taboada, E.; Rodríguez-Fornés, F.; Barba, M.; Dávila, D.; Salvador-Tormo, N.; Guzmán, M.; et al. Targeting Glioma Initiating Cells with A combined therapy of cannabinoids and temozolomide. *Biochem. Pharmacol.* **2018**, *157*, 266–274, doi:10.1016/j.bcp.2018.09.007.
18. López-Valero, I.; Torres, S.; Salazar-Roa, M.; García-Taboada, E.; Hernández-Tiedra, S.; Guzmán, M.; Sepúlveda, J.M.; Velasco, G.; Lorente, M. Optimization of a preclinical therapy of cannabinoids in combination with temozolomide against glioma. *Biochem. Pharmacol.* **2018**, *157*, 275–284, doi:10.1016/j.bcp.2018.08.023.
19. Aparicio-Blanco, J.; Sebastián, V.; Benoit, J.-P.; Torres-Suárez, A. Lipid nanocapsules decorated and loaded with cannabidiol as targeted prolonged release carriers for glioma therapy: In vitro screening of critical parameters. *Eur. J. Pharm. Biopharm.* **2019**, *134*, 126–137, doi:10.1016/j.ejpb.2018.11.020.
20. Kosgodage, U.S.; Uysal-Onganer, P.; MacLatchy, A.; Mould, R.; Nunn, A.V.; Guy, G.W.; Kraev, I.; Chatterton, N.P.; Thomas, E.L.; Inal, J.M.; et al. Cannabidiol Affects Extracellular Vesicle Release, miR21 and miR126, and Reduces Prohibitin Protein in Glioblastoma Multiforme Cells. *Transl. Oncol.* **2019**, *12*, 513–522, doi:10.1016/j.tranon.2018.12.004.
21. Ivanov VN.; Wu J.; Wang TJ.; Hei TK., Inhibition of ATM kinase upregulates levels of cell death induced by cannabidiol and γ-irradiation in human glioblastoma cells. Oncotarget. **2019**, *10*(*8*), 825.

Table S2. CBD's effect on Breast Cancer.

Paper	Breast Cancer cell line	IC_{50} (μM)	Effect on cancer cells	Mechanism of action	Tested in cell line?	Tested in mouse model?
Ligresti et al. (2006)[1]	MCF-7<br>MDA-MB-231	8.2 ± 0.3<br>10.6 ± 1.8<br>*	Anti-proliferative<br>Cell-cycle arrest (increases in G_1, S) (MCF-7 specific)<br>Apoptosis (MDA-MB-231 specific)<br>Reduces tumor size/volume<br>Reduces in number of lung metastasis	Activates CB_2 and TRPV1<br>Generates ROS	Yes	Yes
Sultan et al. (2018) [2]	T-47D<br>MDA-MB-231	5<br>2.2<br>**	Anti-proliferative<br>Induces apoptosis	Downregulates of mTOR and Cyclin D1<br>Upregulates PPARγ protein levels and nuclear localization	Yes	No
Elbaz et al. (2015) [3]	SUM159<br>SCP2<br>4T1.2<br>MDA-MB-231<br>MVT-1	NS<br>DDR (SUM159, SCP2, and 4T1.2)<br>**	Anti-proliferative<br>Inhibits migration of cells<br>Inhibits invasive activity<br>Inhibits tumor growth (reduction in size/volume/weight)<br>Inhibits tumor vascularization	Suppresses activation of EGF/EGFR signal transduction pathways<br>Inhibits activation of EGFR, AKT, p42/44 MAPKand NF-kB<br>Inhibits secretion of MMP, expression of phalloidin and actin stress fiber	Yes	Yes

			Reduction in lung metastasis (number and volume)	Decreases recruitment of macrophages to tumor through decreased microenvironmental changes (decreased GM-CSF, CCL3 and MIP-2 levels)		
McAllister et al. (2007) [4]	MDA-MB-231<br>MDA-MB-231-Id-1<br>MDA-MB-436	1.3 (1.0-1.9)<br><br>1.6 (1.1-2.2)<br>**	Anti-proliferative<br>Reduces invasion of cells	Inhibits Id-1 (inhibiting transcription at the promoter level)	Yes	No
McAllister et al. (2011) [5]	MDA-MB-231<br>4T1	NT<br>1.5 (1.3-1.7)<br>**	Anti-proliferative<br>Reduces invasion of cells<br>Cell cycle arrest (increases G_0 and G_1 cells while decreasing S cells)<br>Reduces tumor size (not weight)<br>Reduces number of lung metastases (specifically >2 mm)	Inhibits Id-1 gene expression, proliferation and invasion (mouse model)<br>Modulates ERK and ROS, which both decrease Id-1 expression	Yes	Yes
Shrivastava et al. (2011) [6]	MCF-7<br>ZR-75-1<br>MDA-MB-231<br>SK-BR-3<br><br>MCF-10A = control	NS<br>DDR<br>(Estrogen receptor + and - cells)<br>Little effect<br>***	Anti-proliferative<br>Apoptosis<br>Autophagy	Independent of CB_1, CB_2, and vanilloid<br>Initiates internal (t-BID translocation) and external (Fas-L death receptor engagement) stimuli leading to mitochondrial-mediated apoptosis<br>ER stress leads to increased autophagy<br>Generates ROS<br>Inhibits AKT/mTOR signaling leading to apoptosis and autophagy<br>Balance between autophagy and apoptosis though cleavage and translocation of beclin-1	Yes	No
Kosgodage et al. (2018) [7]	MD-MB-231	NS<br>DDR<br>**<br>****	Anti-proliferative<br>Increases cisplatin-mediated apoptosis/sensitization of cells to chemotherapy	Inhibits EMV, MV (100-200nm), and particularly exosome release<br>Modulates mitochondrial function<br>Decreases expression	Yes	No

				of mitochondrial-associated proteins prohibitin and STAT3		
Murase et al. (2014) [8]	MDA-MB-231<br>MDA-MB-231-Id-1<br>MDA-MB-231-luc-D3H2LN	1.9 (1.5-2.5)<br>2.8<br>1.8 (1.2-2.7)<br>**	Anti-proliferative<br>Anti-metastatic activity (reduced number, cell proliferation, and growth)<br>Prolongs survival	Produces ROS<br>Downregulates Id1	Yes	Yes
Ward et al. (2013) [9]	4T1<br>4T1<br>LN 231<br>MDA-MB-231-luc-D3H2LN	2.7<br>4.1<br>**	Protective against paclitaxel-induced neurotoxicity<br>Synergistic inhibition of viability with paclitaxel	Induces 5-HT1A receptor system	Yes	Yes

ND: Not determined, NS: Not stated but viability tested, DDR: Dose dependent response.

* Crystal Violet Staining, ** MTT cell proliferation assay, *** MTS viability assay, **** EasyCyte 8HT flow cytometer and ViaCount assay.

1. Ligresti, A.; Moriello, A.S.; Starowicz, K.; Matias, I.; Pisanti, S.; De Petrocellis, L.; Laezza, C.; Portella, G.; Bifulco, M.; Di Marzo, V. Antitumor Activity of Plant Cannabinoids with Emphasis on the Effect of Cannabidiol on Human Breast Carcinoma. *J. Pharmacol. Exp. Ther.* **2006**, *318*, 1375–1387, doi:10.1124/jpet.106.105247.
2. Sultan, A.S.; Marie, M.A.; Sheweita, S.A. Novel mechanism of cannabidiol-induced apoptosis in breast cancer cell lines. *Breast* **2018**, *41*, 34–41, doi:10.1016/j.breast.2018.06.009.
3. Elbaz, M.; Nasser, M.W.; Ravi, J.; Wani, N.A.; Ahirwar, D.; Zhao, H.; Oghumu, S.; Satoskar, A.R.; Shilo, K.; Carson, W.E.; et al. Modulation of the tumor microenvironment and inhibition of EGF/EGFR pathway: Novel anti-tumor mechanisms of Cannabidiol in breast cancer. *Mol. Oncol.* **2015**, *9*, 906–919, doi:10.1016/j.molonc.2014.12.010.
4. McAllister, S.D.; Christian, R.T.; Horowitz, M.P.; Garcia, A.; Desprez, P.-Y. Cannabidiol as a novel inhibitor of Id-1 gene expression in aggressive breast cancer cells. *Mol. Cancer Ther.* **2007**, *6*, 2921–2927, doi:10.1158/1535-7163.mct-07-0371.
5. McAllister, S.D.; Murase, R.; Christian, R.T.; Lau, D.; Zielinski, A.J.; Allison, J.; Almanza, C.; Pakdel, A.; Lee, J.; Limbad, C.; et al. Pathways mediating the effects of cannabidiol on the reduction of breast cancer cell proliferation, invasion, and metastasis. *Breast Cancer Res. Treat.* **2011**, *129*, 37–47, doi:10.1007/s10549-010-1177-4.
6. Shrivastava, A.; Kuzontkoski, P.M.; Groopman, J.E.; Prasad, A. Cannabidiol Induces Programmed Cell Death in Breast Cancer Cells by Coordinating the Cross-talk between Apoptosis and Autophagy. *Mol. Cancer Ther.* **2011**, *10*, 1161–1172, doi:10.1158/1535-7163.mct-10-1100.
7. Kosgodage, U.S.; Mould, R.; Henley, A.B.; Nunn, A.V.; Guy, G.W.; Thomas, E.L.; Inal, J.M.; Bell, J.D.; Lange, S. Cannabidiol (CBD) Is a Novel Inhibitor for Exosome and Microvesicle (EMV) Release in Cancer. *Front. Pharmacol.* **2018**, *9*, 889, doi:10.3389/fphar.2018.00889.

8. Murase, R.; Kawamura, R.; Singer, E.; Pakdel, A.; Sarma, P.; Judkins, J.; Elwakeel, E.; Dayal, S.; Martínez-Martínez, E.; Amere, M.; et al. Targeting multiple cannabinoid anti-tumour pathways with a resorcinol derivative leads to inhibition of advanced stages of breast cancer. *Br. J. Pharmacol.* **2014**, *171*, 4464–4477, doi:10.1111/bph.12803.
9. Ward, S.J.; McAllister, S.D.; Kawamura, R.; Murase, R.; Neelakantan, H.; A Walker, E. Cannabidiol inhibits paclitaxel-induced neuropathic pain through 5- HT 1A receptors without diminishing nervous system function or chemotherapy efficacy. *Br. J. Pharmacol.* **2014**, *171*, 636–645, doi:10.1111/bph.12439.

10. **Table S3.** CBD's Effect on Lung Cancer.

Paper	Lung Cancer cell line	IC_{50} (μM)	Effect on cancer cells	Mechanism of action	Tested in cell line?	Tested in mouse model?
Ramer et al. (2010) [1]	A549	ND	Anti-invasive<br>Anti-metastatic	Induces TIMP-1	Yes	Yes
Ramer et al. (2010) [2]	A549<br>H460<br>H358	ND	Anti-invasive (independent of drug toxicity)	Downregulates PAI-1<br>Dependent on CB_1, CB_2 and TRPV1	Yes	Yes
Ramer et al. (2012) [3]	A549<br>H460	ND	Anti-invasive<br>Anti-metastatic	Activates p42/44 MAPK<br>Increases downstream ICAM-1 prior to TIMP-1 induction<br>Dependent on CB_1, CB_2 and TRPV1	Yes	Yes
Ramer et al. (2013) [4]	A549<br>H460<br>Primary lung tumor cells obtained from a patient with additional brain metastasis	3.47<br>2.80<br>0.124 *	Apoptosis	Induces COX-2 and its derived products PGD_2 and 15d-PGJ_2<br>Activates PPAR-γ	Yes	Yes
Haustein et al. (2014) [5]	A549<br>H460<br>Brain metastatic cells from patient with primary lung tumor<br>BEAS-2B (control)	ND	Anti-invasive<br>Adhesion	Increases LAK cell-mediated tumor cell lysis through increased ICAM-1<br>Dependent on CB_1, CB_2 and TRPV1	Yes	Yes

ND: Not determined.

* WST-1 assay.

1. Ramer, R.; Merkord, J.; Rohde, H.; Hinz, B. Cannabidiol inhibits cancer cell invasion via upregulation of tissue inhibitor of matrix metalloproteinases-1. *Biochem. Pharmacol.* **2010**, *79*, 955–966, doi:10.1016/j.bcp.2009.11.007.
2. Ramer, R.; Rohde, A.; Merkord, J.; Rohde, H.; Hinz, B. Decrease of Plasminogen Activator Inhibitor-1 May Contribute to the Anti-Invasive Action of Cannabidiol on Human Lung Cancer Cells. *Pharm. Res.* **2010**, *27*, 2162–2174, doi:10.1007/s11095-010-0219-2.
3. Ramer, R.; Bublitz, K.; Freimuth, N.; Merkord, J.; Rohde, H.; Haustein, M.; Borchert, P.; Schmuhl, E.; Linnebacher, M.; Hinz, B. Cannabidiol inhibits lung cancer cell invasion and metastasis via intercellular adhesion molecule-1. *FASEB J.* **2011**, *26*, 1535–1548, doi:10.1096/fj.11-198184.

4. Ramer, R.; Heinemann, K.; Merkord, J.; Rohde, H.; Salamon, A.; Linnebacher, M.; Hinz, B. COX-2 and PPAR- Confer Cannabidiol-Induced Apoptosis of Human Lung Cancer Cells. *Mol. Cancer Ther.* **2012**, *12*, 69–82, doi:10.1158/1535-7163.mct-12-0335.
5 . Haustein, M.; Ramer, R.; Linnebacher, M.; Manda, K.; Hinz, B. Cannabinoids increase lung cancer cell lysis by lymphokine-activated killer cells via upregulation of ICAM-1. *Biochem. Pharmacol.* **2014**, *92*, 312–325, doi:10.1016/j.bcp.2014.07.014.

Table S4. CBD's effect on Colorectal Cancers.

**Paper**	**CRC cell line**	**IC_{50} (μM)**	**Effect on cancer cells**	**Mechanism of action**	**Tested in cell line?**	**Tested in mouse model?**
Sreevalsan et al. (2011) [1]	SW480	5.95 after 2 days<br>5.06 after 3 days<br>**	Anti-proliferative<br>Apoptosis	Induces phosphatases<br>Dependent on CB_1 and CB_2	Yes	No
Aviello et al. (2012) [2]	Caco-2<br>HCT116	0.67 (0.0145-31.4)<br>*	Anti-proliferative<br>Reduces aberrant crypt foci, polyps, and tumors.	Downregulates pAKT<br>Upregulates caspase-3<br>Protects against oxidative damage<br>Dependent on CB_1, TRPV1 and PPARγ	Yes	Yes
Macpherson et al. (2014) [3]	Caco-2	ND	Anti-proliferative	Induces ROS production		
Raup-Konsavage et al. (2018) [4]	SW480<br>SW620<br>HT-29<br>DLD-1<br>HCT116<br>LS174<br>RKO<br>CCD 841 CoTr (Control)	16.4 ± 2.5<br>ND<br>19.8 ± 4.4<br>19.8 ± 1.4<br>ND<br>ND<br>ND<br>*	Anti-proliferative<br>Chemoprotective effect	Reduces Wnt/*B*-catenin pathway before translocation of *B*-catenin	Yes	No
Jeong et al. (2019) [5]	DLD-1<br>Colo205 (oxaliplatin resistant strains)	NS<br>DDR<br>***	*CBD + Oxaliplatin:*<br>Autophagy	Produces NOS3<br>Induces ROS (including mitochondrial ROS)<br>Induces mitochondrial dysfunction via reduced SOD2 expression	Yes	Yes
Jeong et al. (2019) [6]	DCRC<br>HCT116<br>DLD-1<br>CCD-18co	ND<br>***	Apoptosis<br>Decreases tumor size	Induces Noxa activation through:<br>Increased ROS<br>Increased ER stress	Yes	Yes

Kim et al. (2019) [7]	(control)<br>HCT116<br>HT26<br>DLD-1<br>Control:<br>CCD18CO	NS<br>DDR<br>*	*CBD + TRAIL:*<br>Apoptosis<br>Decreased tumor growth	Activates CHOP vis ROS-induced ER stress<br>Increases DR5 via ER stress	Yes	Yes

ND: Not determined, NS: Not stated, DDR: Dose dependent response.

* MTTassay, **Cells counted for IC50 ***WST-1 assay.

1. Sreevalsan, S.; Joseph, S.; Jutooru, I.; Chadalapaka, G.; Safe, S. Induction of apoptosis by cannabinoids in prostate and colon cancer cells is phosphatase dependent. *Anticancer. Res.* 2011, *31*, 3799–807.
2. Aviello, G.; Romano, B.; Borrelli, F.; Capasso, R.; Gallo, L.; Piscitelli, F.; Di Marzo, V.; Izzo, A.A. Chemopreventive effect of the non-psychotropic phytocannabinoid cannabidiol on experimental colon cancer. *J. Mol. Med.* **2012**, *90*, 925–934, doi:10.1007/s00109-011-0856-x.
3 . MacPherson, T.; Armstrong, J.A.; Criddle, D.N.; Wright, K.L. Physiological intestinal oxygen modulates the Caco-2 cell model and increases sensitivity to the phytocannabinoid cannabidiol. *Vitr. Cell. Dev. Biol. - Anim.* **2014**, *50*, 417–426, doi:10.1007/s11626-013-9719-9.
4. Raup-Konsavage, W.M.; Johnson, M.; Legare, C.A.; Yochum, G.S.; Morgan, D.J.; Vrana, K.E. Synthetic Cannabinoid Activity Against Colorectal Cancer Cells. *Cannabis Cannabinoid Res.* **2018**, *3*, 272–281, doi:10.1089/can.2018.0065.
5. Jeong, S.; Kim, B.G.; Kim, D.Y.; Kim, B.R.; Kim, J.L.; Park, S.H.; Na, Y.J.; Jo, M.J.; Yun, H.K.; Jeong, Y.A.; et al. Cannabidiol Overcomes Oxaliplatin Resistance by Enhancing NOS3- and SOD2-Induced Autophagy in Human Colorectal Cancer Cells. *Cancers* **2019**, *11*, 781, doi:10.3390/cancers11060781.
6. Jeong, S.; Yun, H.K.; Jeong, Y.A.; Jo, M.J.; Kang, S.H.; Kim, J.L.; Kim, D.Y.; Park, S.H.; Kim, B.R.; Na, Y.J.; et al. Cannabidiol-induced apoptosis is mediated by activation of Noxa in human colorectal cancer cells. *Cancer Lett.* **2019**, *447*, 12–23, doi:10.1016/j.canlet.2019.01.011.
7. Kim, J.L.; Kim, B.R.; Kim, D.Y.; Jeong, Y.A.; Jeong, S.; Na, Y.J.; Park, S.H.; Yun, H.K.; Jo, M.J.; Kim, B.G.; et al. Cannabidiol Enhances the Therapeutic Effects of TRAIL by Upregulating DR5 in Colorectal Cancer. *Cancers* **2019**, *11*, 642, doi:10.3390/cancers11050642.

Table S5. CBD's effect on Leukemia and Lymphoma.

**Paper**	**Leukemia/Lymphoma cell line**	**IC_{50} (μM)**	**Effect on cancer cells**	**Mechanism of Action**	**Tested in cell line?**	**Tested in mouse model?**
Gallily et al. (2003) [1]	<u>Leukemia</u><br>HL-60	ND	*CBD + γ irradiation:*<br>Apoptosis	Activates caspase-3	Yes	No
Ligresti et al. (2006) [2]	<u>Leukemia</u><br>RBL-2H3	6.3 ± 1.5<br>*	ND	ND	Yes	No
McKallip et al. (2006) [3]	<u>Lymphoma</u><br>EL-4<br><u>Leukemia</u><br>Jurkat<br>MOLT-4	ND<br>****	Anti-proliferative<br>Apoptosis<br>Decreases tumor burden	Independent of CB_1 and TRPV1<br>Dependent on CB_2<br>Induces ROS<br>Reduces mitochondrial membrane potential<br>Induces cleavage of capsase-8 and	Yes	Yes

				Bid, and reduction of procaspase-2, -9, -10<br>Increases cytosolic cytochrome C<br>Increases Nox4 and p22^{phox}<br>Reduces p-p38		
Lee et al. (2008) [4]	<u>Lymphoma</u><br>EL-4<br>Murine thymocytes	10.75 ± 1.35<br>7.01 ± 0.31	Apoptosis	Induces ROS<br>Decreases cellular thiols	Yes	No
Scott et al. (2017) [5]	<u>Leukemia</u><br>CEM<br>HL-60	7.8 ± 0.21<br>12<br>**	*CBD + Δ⁹-THC or CBG + VIN or CYT*<br>Anti-proliferative<br>Apoptosis<br>Decreases dose of chemotherapies required<br>Killing effect is sequence dependent (chemo then phytocannabinoids)	ND	Yes	No
Kalenderoglou et al. (2017) [6]	<u>T-ALL</u><br>Jurkat	6.4 ± 2.9 (5% serum)<br>2.5 ± 0.2 (1% serum)<br>43.4 ± 5.1 (12% O_2)<br>****<br>*****	Anti-proliferative<br>Induces cell cycle arrest (G1 increase)<br>Increases number of small cells<br>Additive effect with Doxorubicin	Reduces pPKB/pAkt and ribosomal protein S6	Yes	No
Olivas-Aguirre et al. (2019) [7]	<u>T-ALL</u><br>Jurkat<br>MOLT-3<br>CCFR-CEM<br><u>B-ALL</u><br>Reh<br>RS4;11<br><u>CML</u><br>K562	ND<br>***	<u>T-ALL cells</u><br>At low concentrations, increases proliferation and autophagy<br>Anti-proliferative at higher concentrations<br>Inhibits migration<br>Apoptosis and early necrosis<br>Changes cell	Targets mitochondria in T-ALL<br>Induces ROS<br>Reduces mitochondrial transmembrane potential<br>Increases Cytochrome-C release<br>Induces mitochondrial	Yes	No

			morphology	Ca^{2+} overload from intracellular stores via mitochondrial Ca^{2+} uniporter<br>Induces mitochondrial permeability transition pore opening and subsequent Ca^{2+} release from the ER<br>Independent of CB_1, CB_2, and GRP55<br>In 3D analysis of CBD interacting with voltage-dependent anion channel, interacts with Thr9, Asp12, and Leu13 residues at the N-terminus and neighboring pore residues Val146, Gln157, Gly175, Gln182, and His184		
Togano et al. (2019) [8]	<u>Burkitt's Lymphoma</u><br>Jiyoye<br>Mutu I	ND<br>*****	Enhances cell-mediated cytotoxicity	Increases ICAM-1<br>Attenuates lymphokine-activated killer cell resistance to cell-mediated toxicity by AF1q via ICAM-1	Yes	No

ND: Not determined.

Viability markers: *Crystal Violet vital staining + spectrophotometer analysis, ** MTT-based assay, *** resazurin-based metabolic assay, ****Trypan blue dye exclusion, *****PrestoBlue.

CYT: cytarabine, VIN: vincristine.

CBG: cannabigerol.

ER: endoplasmic reticulum.

T-ALL: *T*-cell acute lymphoblastic leukemia, B-ALL: *B*-cell acute lymphoblastic leukemia, CML: Chronic myelogenous leukemia.

1. Gallily, R.; Even-Chena, T.; Katzavian, G.; Lehmann, D.; Dagan, A.; Mechoulam, R. γ-Irradiation Enhances Apoptosis Induced by Cannabidiol, a Non-psychotropic Cannabinoid, in Cultured HL-60 Myeloblastic Leukemia Cells. *Leuk. Lymphoma* **2003**, *44*, 1767–1773, doi:10.1080/1042819031000103917.
2. Ligresti, A.; Moriello, A.S.; Starowicz, K.; Matias, I.; Pisanti, S.; De Petrocellis, L.; Laezza, C.; Portella, G.; Bifulco, M.; Di Marzo, V. Antitumor Activity of Plant Cannabinoids with Emphasis on the Effect of Cannabidiol on Human Breast Carcinoma. *J. Pharmacol. Exp. Ther.* **2006**, *318*, 1375–1387, doi:10.1124/jpet.106.105247.
3 . McKallip, R.J.; Jia, W.; Schlomer, J.; Warren, J.W.; Nagarkatti, P.S.; Nagarkatti, M. Cannabidiol-Induced Apoptosis in Human Leukemia Cells: A Novel Role of Cannabidiol in the Regulation of p22phox and Nox4 Expression. *Mol. Pharmacol.* **2006**, *70*, 897–908, doi:10.1124/mol.106.023937.
4. Lee, C.-Y.; Wey, S.-P.; Liao, M.-H.; Hsu, W.-L.; Wu, H.-Y.; Jan, T.-R. A comparative study on cannabidiol-induced apoptosis in murine thymocytes and EL-4 thymoma cells. *Int. Immunopharmacol.* **2008**, *8*, 732–740, doi:10.1016/j.intimp.2008.01.018.
5. Scott, K.A.; Dalgleish, A.G.; Liu, W.M. Anticancer effects of phytocannabinoids used with chemotherapy in leukaemia cells can be improved by altering the sequence of their administration. *Int. J. Oncol.* **2017**, *51*, 369–377, doi:10.3892/ijo.2017.4022.
6. Kalenderoglou, N.; MacPherson, T.; Wright, K.L. Cannabidiol Reduces Leukemic Cell Size – But Is It Important? *Front. Pharmacol.* **2017**, *8*, doi:10.3389/fphar.2017.00144.
7. Olivas-Aguirre, M.; Torres-López, L.; Valle-Reyes, J.S.; Hernández-Cruz, A.; Pottosin, I.; Dobrovinskaya, O. Cannabidiol directly targets mitochondria and disturbs calcium homeostasis in acute lymphoblastic leukemia. *Cell Death Dis.* **2019**, *10*, 1–19, doi:10.1038/s41419-019-2024-0.
8. Togano, T.; Kim, N.; Kim, N.; Park, G.S.; Park, A.K.; Bennet, M.; Park, J. The evaluation of Cannabidiol's effect on the immunotherapy of Burkitt lymphoma. *Biochem. Biophys. Res. Commun.* **2019**, *520*, 225–230, doi:10.1016/j.bbrc.2019.10.001.

Table S6. CBD's effect on Prostate Cancer.

Paper	Prostate Cancer cell line	IC_{50} (μM)	Effect on cancer cells	Mechanism of Action	Tested in cell line?	Tested in mouse model?
Ligresti et al (2006) [1]	DU-145	20 ± 1.8 *	Anti-proliferative	N.D.	Yes	No
Sreevalsan et al. (2011) [2]	LNCaP	5.95 (2 days)<br>5.06 (3 days)	Anti-proliferative<br>Apoptosis	Caspase dependent<br>Phosphatase dependent (*DUSP1, DUSP4* and *DUSP10 mRNA* induced)<br>Dependent on CB_2	Yes	No
De Petrocellis et al. (2013) [3]	LNCaP<br>22RV1<br>DU-145<br>PC-3	25.0 ± 3 (72h)<br>5.7 ± 2 (16h starved)<br>23.1 ± 7 (72h)<br>8.4 ±2 (16h starved)<br>25.3 ± 8 (72h)<br>5.4 ± 1	Anti-proliferative<br>Apoptosis<br>Inhibits the cell cycle in DU-145 (G1-S transition)<br>*CBD-BDS:*<br>Potentiates effect of bicalutamide and docetaxel<br>Reduces tumor size	Enhances phosphorylation of p38<br>Induces PUMA and CHOP expression (intrinsic apoptosis pathway)<br>Increases intracellular Ca^{2+}<br>Partially dependent on TRPM8 antagonism<br>Downregulates androgen receptor (AR)<br>Downregulates p53 (in AR expressing cells)<br>Induces ROS (in AR	Yes	Yes

		(16h starved)<br>17.4 ± 3 (72h)<br>5.2 ± 1 (16h starved)<br>**		expressing cells)<br>Upregulates $p27^{kip}$<br>G-protein-coupled estrogen receptor dependent		
Sharma et al. (2014) [4]	LNCaP<br>DU-145<br>PC-3	ND<br>***	Anti-proliferative<br>Apoptosis<br>Cell cycle arrest (G0/G1)	Downregulates CB_1, CB_2, AR expression<br>Downregulates PSA, VEGF, IL-6, and IL-8<br>Inhibits spheroid formation (prostate cancer stem cell property)	Yes	No
Kosgodage et al. (2018) [5]	PC-3	ND<br>**	Anti-proliferative	Reduces release of exosomes and mircovesicles<br>Reduces CD63 expression<br>Decreases oxygen consumption rate<br>Reduces prohibitin and STAT3 levels	Yes	No

ND: Not determined.

*Trypan blue dye exclusion method ** MTT test *** MTS.

Starved = absence of serum proteins.

1. Ligresti, A.; Moriello, A.S.; Starowicz, K.; Matias, I.; Pisanti, S.; De Petrocellis, L.; Laezza, C.; Portella, G.; Bifulco, M.; Di Marzo, V. Antitumor Activity of Plant Cannabinoids with Emphasis on the Effect of Cannabidiol on Human Breast Carcinoma. *J. Pharmacol. Exp. Ther.* **2006**, *318*, 1375–1387, doi:10.1124/jpet.106.105247.
2. Sreevalsan, S.; Joseph, S.; Jutooru, I.; Chadalapaka, G.; Safe, S. Induction of apoptosis by cannabinoids in prostate and colon cancer cells is phosphatase dependent. *Anticancer. Res.* 2011, *31*, 3799–807.
3. De Petrocellis L.; Ligresti A.; Schiano Moriello A., et al. Non-THC cannabinoids inhibit prostate carcinoma growth in vitro and in vivo: pro-apoptotic effects and underlying mechanisms. *British journal of pharmacology*. *2013*, *168*(*1*), 79–102.
4. Sharma, M.; Hudson, J.B.; Adomat, H.; Guns, E.S.T.; Cox, M.E. In Vitro Anticancer Activity of Plant-Derived Cannabidiol on Prostate Cancer Cell Lines. *Pharmacol. Pharm.* **2014**, *5*, 806–820, doi:10.4236/pp.2014.58091.
5. Kosgodage, U.S.; Mould, R.; Henley, A.B.; Nunn, A.V.; Guy, G.W.; Thomas, E.L.; Inal, J.M.; Bell, J.D.; Lange, S. Cannabidiol (CBD) Is a Novel Inhibitor for Exosome and Microvesicle (EMV) Release in Cancer. *Front. Pharmacol.* **2018**, *9*, 889, doi:10.3389/fphar.2018.00889.

Table S7. CBD's Effect on Other Cancer Types.

**Paper**	**Cell line**	**IC_{50} (μM)**	**Effect on cancer cells**	**Mechanism of Action**	**Tested in cell line?**	**Tested in mouse model?**
**Cervical Cancer**						
Ramer et al.	HeLa	ND	Anti-proliferative	Dependent on CB_1, CB_2	Yes	No

(2010) [1]	C33A	*	Anti-invasive	and TRPV1<br>Induces TIMP-1<br>Induces p38 and p42/44 MAPK activation		
Lukhele et al. (2016) [2]	HeLa<br>ME-180<br>SiHa	10.18<br>4.77<br>10.18<br>**	Anti-proliferative<br>Apoptosis<br>Changes cell morphology	Decreases ATP<br>Increases caspase 3/7<br>Increases expression of p53 and Bax<br>Decreases expression of RBBP6 and Bcl-2<br>Upregulates caspase-3 and -9	Yes	No
**Hepatocellular Carcinoma**						
Kosgodage et al. (2018) [3]	Hep G2	ND<br>*	Anti-proliferative<br>Sensitizes cells to cisplatin-mediated apoptosis	Reduces release of exosomes and microvesicles<br>Reduces CD63 expression<br>Reduces prohibitin and STAT3 levels	Yes	No
Neumann-Raizel et al. (2019) [4]	BNL1 ME<br>HEK293T	ND<br>***	*CBD + Doxorubicin:*<br>Increases accumulation of doxorubicin in cells<br>Anti-proliferative<br>Increases potency of doxorubicin	Facilitates entry of doxorubicin through TRPV2<br>Prevents doxorubicin clearance through inhibition of P-glycoprotein ATPase transporter	Yes	No
**Thyroid Cancer**						
Ligresti et al. (2006) [5]	KiMol (thyroid)	6.0 ± 3.0<br>****	Anti-proliferative<br>Apoptosis<br>Cell cycle arrest (G_1/S)<br>Decreases tumor size	ND	Yes	Yes
**Renal Cell Carcinoma**						
Taha et al. (2019) [6]	Renal cell carcinoma<br>Non-Small Cell Lung Cancer<br>Melanoma	ND	Decreases response rate of nivolumab	ND	No	No
**Gastric Cancer**						
Ligresti et al. (2006) [5]	AGS	7.5 ± 1.3<br>****	Anti-proliferative	ND	Yes	No
Zhang et al. (2019) [7]	SGC-7901	74.41<br>*****	Anti-proliferative<br>Apoptosis<br>Cell cycle arrest (G0-G1)	Inhibits CDK2 and cyclin E expression<br>Increases ATM and p21 expression<br>Decreases p53 expression	Yes	Yes

Jeong et al. (2019) [8]	AGS<br>MKN45<br>MKN74<br>SNU638<br>NCI-N87	ND<br>*	Anti-proliferative<br>Apoptosis<br>Inhibits tumor growth	Increases cleavage of caspase-3 and -9<br>Increases cytoplasmic cytochrome c, Apaf-1, Bad, and Bax expression<br>Decreases Bcl-2 expression<br>Induces ROS<br>Increases caspase 3/7 activity<br>Mitochondrial dysfunction<br>Suppresses XIAP through the ubiquitin-proteasome system<br>Increases Smac release<br>Elevates ER stress and chaperone proteins	Yes	Yes
**Melanoma**						
Simmerman et al. (2019) [9]	B16F10	ND	Increases survival time and improves quality of life<br>Reduced tumor growth	ND	No	Yes

**Pancreatic Cancer**						
**Ferro et al. (2018)[10]**	**ASPC1<br>HPAFII<br>BXPC3<br>PANC1<br>KPC mice**	**ND**	**Anti-proliferative<br>Independent of apoptosis<br>Cell cycle arrest (G1/S)<br>Increases survival time<br>Inhibits growth**	**Inhibition of GPR55<br>Inhibition of MEK/ERK and ERK-dependent pathways<br>Reduced expression of cyclin D1<br>Activation of tumor suppressor retinoblastoma**	**Yes**	Yes

ND: Not determined.

* WST-1 test **MTT assay ***CellTiter-Fluor Cell Viability Assay ****Crystal violet vital staining ***** CCK-8 assay.

Smac: second mitochondria-derived activator of caspase.

XIAP: X-linked inhibitor of apoptosis.

MMP: mitochondrial membrane potential.

1. Ramer, R.; Merkord, J.; Rohde, H.; Hinz, B. Cannabidiol inhibits cancer cell invasion via upregulation of tissue inhibitor of matrix metalloproteinases-1. *Biochem. Pharmacol.* **2010**, *79*, 955–966, doi:10.1016/j.bcp.2009.11.007.
2. Lukhele, S.T.; Motadi, L.R. Cannabidiol rather than Cannabis sativa extracts inhibit cell growth and induce apoptosis in cervical cancer cells. *BMC Complement. Altern. Med.* **2016**, *16*, 335, doi:10.1186/s12906-016-1280-0.

3. Kosgodage, U.S.; Mould, R.; Henley, A.B.; Nunn, A.V.; Guy, G.W.; Thomas, E.L.; Inal, J.M.; Bell, J.D.; Lange, S. Cannabidiol (CBD) Is a Novel Inhibitor for Exosome and Microvesicle (EMV) Release in Cancer. *Front. Pharmacol.* **2018**, *9*, 889, doi:10.3389/fphar.2018.00889.
4. Neumann-Raizel H.; Shilo A.; Lev S., et al. 2-APB and CBD-mediated targeting of charged cytotoxic compounds into tumor-like cells suggests the involvement of TRPV2 channels. *Frontiers in pharmacology*. **2019**, *10*, 1198.
5. Ligresti, A.; Moriello, A.S.; Starowicz, K.; Matias, I.; Pisanti, S.; De Petrocellis, L.; Laezza, C.; Portella, G.; Bifulco, M.; Di Marzo, V. Antitumor Activity of Plant Cannabinoids with Emphasis on the Effect of Cannabidiol on Human Breast Carcinoma. *J. Pharmacol. Exp. Ther.* **2006**, *318*, 1375–1387, doi:10.1124/jpet.106.105247.
6. Taha, T.; Meiri, D.; Talhamy, S.; Wollner, M.; Peer, A.; Bar-Sela, G. Cannabis Impacts Tumor Response Rate to Nivolumab in Patients with Advanced Malignancies. *Oncol.* **2019**, *24*, 549–554, doi:10.1634/theoncologist.2018-0383.
7. Zhang, X.; Qin, Y.; Pan, Z.; Li, M.; Liu, X.; Chen, X.; Qu, G.; Zhou, L.; Xu, M.; Zheng, Q.; et al. Cannabidiol Induces Cell Cycle Arrest and Cell Apoptosis in Human Gastric Cancer SGC-7901 Cells. *Biomol.* **2019**, *9*, 302, doi:10.3390/biom9080302.
8. Jeong, S.; Jo, M.J.; Yun, H.K.; Kim, D.Y.; Kim, B.R.; Kim, J.L.; Park, S.H.; Na, Y.J.; A Jeong, Y.; Kim, B.G.; et al. Cannabidiol promotes apoptosis via regulation of XIAP/Smac in gastric cancer. *Cell Death Dis.* **2019**, *10*, 1–13, doi:10.1038/s41419-019-2001-7.
9. Simmerman, E.; Qin, X.; Yu, J.C.; Baban, B. Cannabinoids as a Potential New and Novel Treatment for Melanoma: A Pilot Study in a Murine Model. *J. Surg. Res.* **2019**, *235*, 210–215, doi:10.1016/j.jss.2018.08.055.
10. Ferro, R.; Adamska, A.; Lattanzio, R.; Mavrommati, I.; Edling, C.E.; Arifin, S.A.; Fyffe, C.A.; Sala, G.; Sacchetto, L.; Chiorino, G.; et al. GPR55 signalling promotes proliferation of pancreatic cancer cells and tumour growth in mice, and its inhibition increases effects of gemcitabine. *Oncogene* **2018**, *37*, 6368–6382, doi:10.1038/s41388-018-0390-1.
